# *Sall4* regulates posterior trunk mesoderm development by promoting mesodermal gene expression and repressing neural genes in the mesoderm

**DOI:** 10.1242/dev.202649

**Published:** 2024-03-04

**Authors:** Matthew P. Pappas, Hiroko Kawakami, Dylan Corcoran, Katherine Q. Chen, Earl Parker Scott, Julia Wong, Micah D. Gearhart, Ryuichi Nishinakamura, Yasushi Nakagawa, Yasuhiko Kawakami

**Affiliations:** ^1^ Department of Genetics, Cell Biology and Development, University of Minnesota, Minneapolis, MN 55455, USA; ^2^ Stem Cell Institute, University of Minnesota, Minneapolis, MN 55455, USA; ^3^ Developmental Biology Center, University of Minnesota, Minneapolis, MN 55455, USA; ^4^ Department of Neuroscience, University of Minnesota, Minneapolis, MN 55455, USA; ^5^ Department of Obstetrics, Gynecology and Women's Health, University of Minnesota, Minneapolis, MN 55455, USA; ^6^ Department of Kidney Development, Institute of Molecular Embryology and Genetics, Kumamoto University, Kumamoto 860-0811, Japan

**Keywords:** Posterior trunk, Presomitic mesoderm, Somites, *Sall4*, WNT signaling, Chromatin accessibility

## Abstract

The trunk axial skeleton develops from paraxial mesoderm cells. Our recent study demonstrated that conditional knockout of the stem cell factor *Sall4* in mice by *TCre* caused tail truncation and a disorganized axial skeleton posterior to the lumbar level. Based on this phenotype, we hypothesized that, in addition to the previously reported role of *Sall4* in neuromesodermal progenitors, *Sall4* is involved in the development of the paraxial mesoderm tissue. Analysis of gene expression and SALL4 binding suggests that *Sall4* directly or indirectly regulates genes involved in presomitic mesoderm differentiation, somite formation and somite differentiation. Furthermore, ATAC-seq in *TCre*; *Sall4* mutant posterior trunk mesoderm shows that *Sall4* knockout reduces chromatin accessibility. We found that *Sall4*-dependent open chromatin status drives activation and repression of WNT signaling activators and repressors, respectively, to promote WNT signaling. Moreover, footprinting analysis of ATAC-seq data suggests that *Sall4*-dependent chromatin accessibility facilitates CTCF binding, which contributes to the repression of neural genes within the mesoderm. This study unveils multiple mechanisms by which *Sall4* regulates paraxial mesoderm development by directing activation of mesodermal genes and repression of neural genes.

## Teaser

**Summary:** *Sall4* regulates posterior trunk mesoderm development by promoting mesodermal gene expression while negatively regulating expression of WNT signaling repressors and neural genes within the mesoderm.

## INTRODUCTION

The vertebrate body progressively develops from the anterior to the posterior during body elongation, in which new tissues are generated at the posterior end during the post-gastrulation stages (Henrique et al., 2015). Among mesodermal tissues generated during body elongation of the embryo, the paraxial mesoderm (PM) is located lateral to the neural tube and notochord. Complex genetic systems regulate PM development. In the posterior part of the PM, the tissue is unsegmented and is called the presomitic mesoderm (PSM). Two transcription factor genes, *Msgn1* and *Tbx6*, act as crucial regulators of PM development. *Msgn1* acts as a master regulator of PM development (Chalamalasetty et al., 2014; Yoon and Wold, 2000). *Tbx6* represses *Sox2* to inhibit neural development and specify the PM from bi-potential neuromesodermal progenitors (Chapman and Papaioannou, 1998; Takemoto et al., 2011). The PSM is more mature in the anterior, and periodic segmentation of the anterior end of the PSM leads to somite formation. This process is controlled by an interaction between the segmentation clock and the maturation wavefront (Hubaud and Pourquié, 2014). Specifically, *Hes7* plays a central role in generating an oscillatory cycle of Notch signaling, which forms the segmentation clock (Bessho et al., 2003). In the wavefront system, a posterior-high to anterior-low gradient of WNT/β-catenin and fibroblast growth factor (FGF) signaling define specific stages of maturation of the PSM (Dequeant and Pourquié, 2008; Kageyama et al., 2012). The arrest of oscillation at the wavefront leads to boundary formation at the anterior edge of the PSM (Maroto et al., 2012; Pourquié, 2011). During a somite cycle, a complex regulatory system that involves Notch signaling, *Tbx6*, *Mesp2* and *Ripply2,* regulates precise formation of the somite boundary during somitogenesis (Chan et al., 2007; Yasuhiko et al., 2006; Zhao et al., 2015).

Somites are initially generated as epithelial spheres, consisting of multipotent progenitor cells, which subsequently undergo morphogenetic changes (Brent and Tabin, 2002; Christ et al., 2007). On the ventral side, cells undergo an epithelial-to-mesenchymal transition to form the sclerotome, and subsequently migrate to form the vertebrae primordium. On the dorsal side, the cells in the dermomyotome edges transition to form the myotome (Gros et al., 2004), which contributes to skeletal muscle of the trunk and limbs. During somite differentiation, several genes are well characterized as specific musculoskeletal lineage markers. Among them, *Pax9* is a chondrogenic lineage marker and *Pax3* is a dermomyotomal myogenic marker (Goulding et al., 1994; Peters et al., 1999; Williams and Ordahl, 1994). Other PM/somite markers include *Tcf15* (also known as *Paraxis*), which is required for somite epithelialization (Burgess et al., 1996). *Meox1* is involved in somite morphogenesis, patterning and differentiation, particularly of sclerotome-derived structures (Mankoo et al., 2003; Skuntz et al., 2009). *Aldh1a2* (also known as *Raldh2*) encodes a rate-limiting enzyme in the retinoic acid synthesis pathway. Retinoic acid, which is secreted from the somite, regulates forelimb initiation, somitogenesis and trunk neurogenesis (Cunningham et al., 2013; Diez del Corral et al., 2002; Dubrulle et al., 2001; Zhao et al., 2009).

*Sall4* encodes a zinc-finger transcription factor (de Celis and Barrio, 2009; Sweetman and Münsterberg, 2006) that is highly and broadly expressed in the posterior part of the mouse embryo (Akiyama et al., 2015; Kohlhase et al., 2002; Tahara et al., 2018). Conditional knockout (cKO) of *Sall4* in the mesoderm lineage and neuromesodermal progenitors using *TCre* (or *Brachyury-Cre*) causes tail truncation. Our previous study demonstrated that *Sall4* is required for neuromesodermal progenitor maintenance and its differentiation into the mesodermal lineage at the expense of neural differentiation (Tahara et al., 2019). The *TCre; Sall4* cKO mutants also exhibited disorganized vertebrae, posterior to the lumbar level (Akiyama et al., 2015; Tahara et al., 2019), which suggests a requirement of *Sall4* for multiple aspects of the development of the axial skeleton in the posterior trunk and tail. For example, *Sall4* may be involved in cell differentiation in the PSM, in somitogenesis and/or in differentiation of somites. However, our previous study focused on neuromesodermal progenitors, and roles of *Sall4* in PM development remain unknown.

In this study, we have examined the expression patterns of genes involved in PM development in the posterior trunk in wild type (WT) and *TCre*; *Sall4* cKO embryos. By revisiting our SALL4 ChIP-seq data (Tahara et al., 2019), we observed SALL4 enrichment in some genes involved in PM development and somite development. Moreover, ATAC-seq analysis of mesoderm tissue from the posterior trunk suggests that SALL4 promotes WNT/β-catenin signaling through regulating accessibility of a set of genes that modulates WNT/β-catenin signaling. Furthermore, our data suggest that *Sall4* regulation of chromatin accessibility negatively regulates neural gene expression in the mesoderm. Taken together, our study supports the idea that *Sall4* regulates posterior trunk mesoderm development through directly promoting transcription of mesodermal genes while inhibiting genes for WNT/β-catenin signaling repression and neural differentiation in the mesoderm.

## RESULTS

### *Sall4* expression in the posterior trunk and tail

**Fig. 1.**
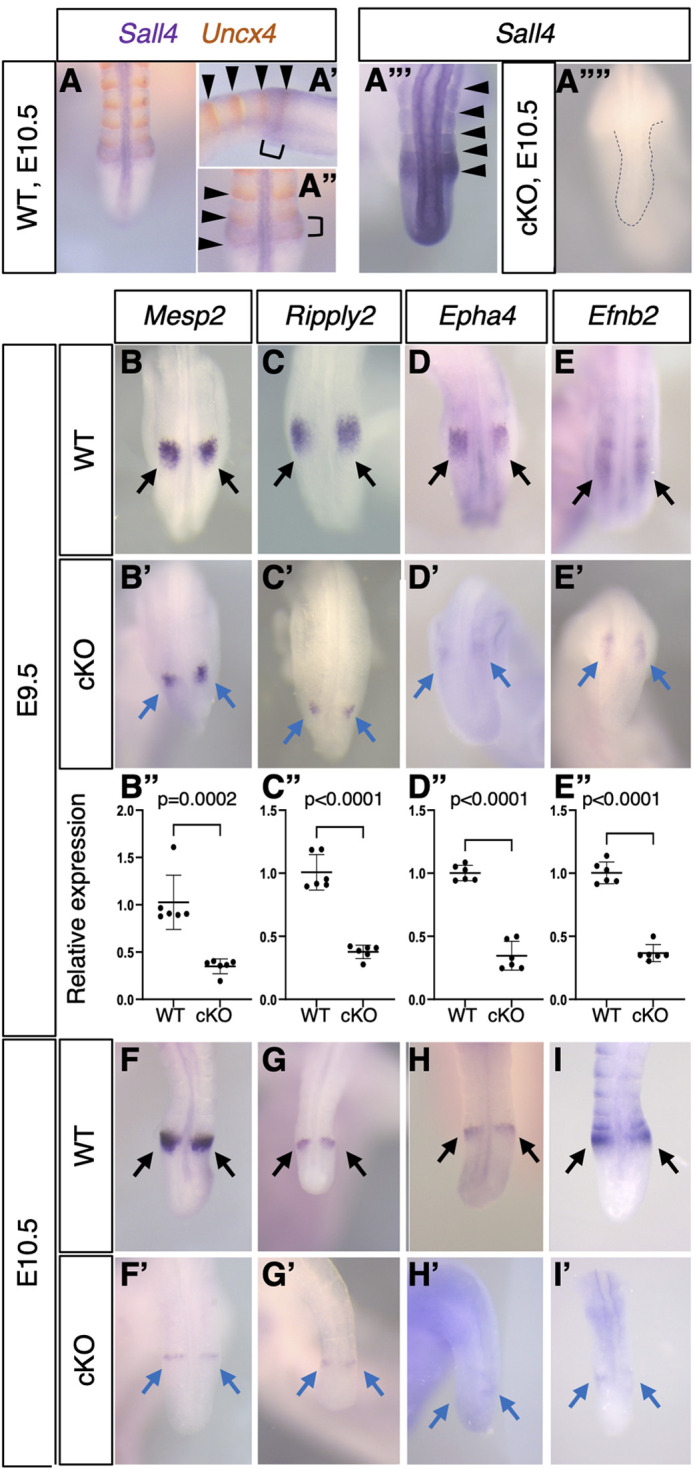
**Gene expression patterns in the anterior part of PSM.** (A-A″) Double color *in situ* hybridization of *Uncx4* (orange) and *Sall4* (purple) at E10.5 (*n*=3). The *Sall4* detection reaction was stopped early to visualize a strong expression domain. A and A″ show dorsal views; A′ shows a lateral view. A′ and A″ show a more-detailed view of the somite-PSM boundary region in A. Arrowheads in A′ and A″ indicate the Uncx4 expression domain. Brackets in A′ and A″ indicate the next somite-forming region at the anterior PSM. (A‴,A⁗) *Sall4 in situ* hybridization in WT (A‴, *n*>30) and *Sall4* cKO (A⁗, *n*=3) embryos at E10.5. Arrowheads in A‴ indicate the *Sall4* expression domain. The dotted line in A⁗ depicts the tail morphology. (B-E′,F-I′) Expression pattern of *Mesp2* [B (*n*=6), B′ (*n*=4), F (*n*=5), F′(*n*=3)], *Ripply2* [C (*n*=6), C′ (*n*=5), G (*n*=6), G′ (*n*=6)], *Epha4* [D (*n*=6), D′ (*n*=4), H (*n*=6), H′ (*n*=5)] and *Efnb2* [E (*n*=6), E′ (*n*=4), I (*n*=6), I′ (*n*=4)] at E9.5 (B-E′) and E10.5 (F-I′). B-E and F-I show dorsal views of the posterior part of the body or tail in wild-type embryos. B′-E′ and F′-I′ show equivalent regions in *Sall4* cKO embryos. Black and blue arrows indicate normal expression in wild type and reduced expression in mutants, respectively. B-E′ and F-I′ are at the same scale. (B″-E″) Relative expression levels of *Mesp2* (B″), *Ripply2* (C″), *Epha4* (D″) and *Efnb2* (E″) in the posterior trunk of wild-type and *Sall4* cKO embryos at E9.5. Each dot represents an embryo. *P* values were obtained using an unpaired *t*-test.

Several studies, including ours, reported the expression pattern of *Sall4* during mouse embryonic development by mRNA *in situ* hybridization. Although *Sall4* is broadly expressed in the posterior part of embryos at E9.5-E10.5, including the entire PSM and the somites, *Sall4* exhibits a particularly strong expression domain around the PSM-somite boundary (Kohlhase et al., 2002; Tahara et al., 2018). In order to characterize this expression domain in more detail, we performed *Sall4* and *Uncx4* double *in situ* hybridization (Fig. 1A). *Uncx4* is expressed in the posterior part of the somite and in the area S0, where the somite is forming at the anterior edge of the PSM (Mansouri et al., 1997). Strong *Sall4* signals are detected in the entire S0, suggesting that *Sall4* might play a role in somite formation (Fig. 1A′-A″″).

At the anterior edge of the PSM, several genes are involved in the creation of a new pair of somites. We examined the expression pattern of such genes in wild-type and *Sall4* cKO embryos. Although robust recombination by the *TCre* driver occurs as early as E7.5 in cells that contribute to the post-cranial mesoderm tissue (Akiyama et al., 2015; Perantoni et al., 2005), SALL4 protein depletion in the posterior body becomes evident at E9.5 and is more obvious at E10.5 (Tahara et al., 2019) (Fig. S1). Therefore, we examined gene expression at E9.5-E9.75 (24- to 28-somite stage; we refer to this stage as E9.5, for simplicity) and E10.5 (34- to 39-somite stage) by whole-mount *in situ* hybridization. *Mesp2* and *Ripply2* are expressed at the anterior PSM and are involved in somite boundary formation (Biris et al., 2007; Oginuma et al., 2008; Zhao et al., 2015). Expression of both *Mesp2* and *Ripply2* in *Sall4* cKO embryos exhibited comparable spatial distribution to wild type but with reduced signal intensity at E9.5 (Fig. 1B,B′,C,C′). The Eph/ephrin signaling activity participates in the epithelialization of somite boundary at the anterior PSM (Watanabe et al., 2009). In *Sall4* cKO embryos, the expression patterns of both *Epha4* and *Efnb2* were similar to wild type but the signal intensity was reduced at E9.5 (Fig. 1D,D′,E,E′). Quantitative analysis of transcripts in the trunk tissue posterior to the newest somite pair via qRT-PCR confirmed that expression of these genes was reduced in *Sall4* cKO embryos (Fig. 1B″-E″). At E10.5, these genes exhibited considerably reduced signals (Fig. 1F,F′,G,G′,H,H′,I,I′).

To determine whether the expression of the above genes is directly regulated by SALL4, we re-visited our previously published data of SALL4 ChIP-seq using mesoderm tissue from the posterior part of the body at E9.5 (Tahara et al., 2019), which showed that ∼17% of SALL4 binding peaks are located at promoters. SALL4 is enriched around the transcription start site (TSS) to exon 1 of *Mesp2*, *Ripply2*, *Epha4* and *Efnb2* (Fig. S2, Tables S1 and S2), suggesting direct regulation of these genes by *Sall4*.

### *Sall4* regulates differentiation in PSM

**Fig. 2.**
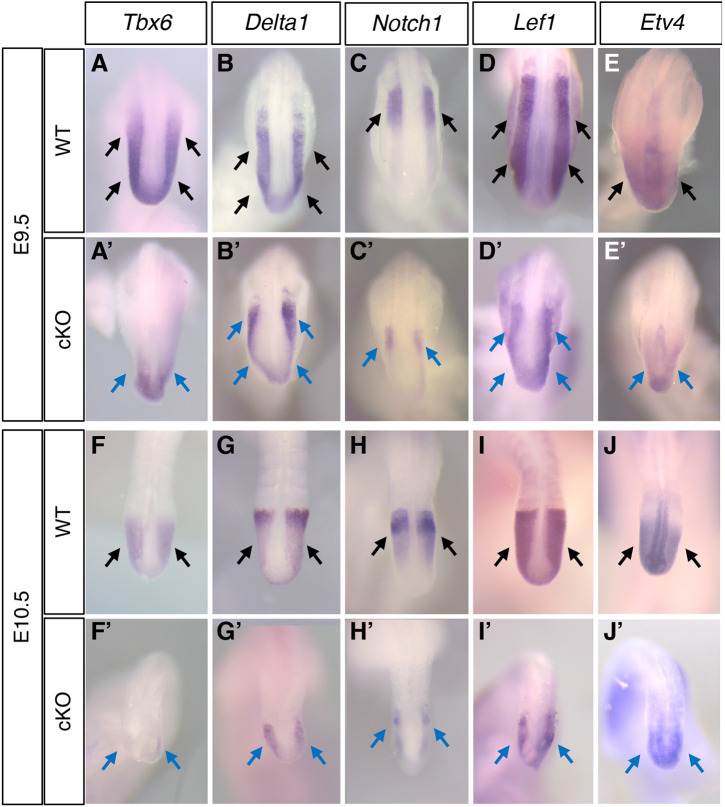
**Gene expression patterns in the PSM.** (A-J′) Expression patterns of *Tbx6* [A (*n*=6), A′ (*n*=5), F (*n*=6), F′ (*n*=6)], *Dll1* [B (*n*=6), B′ (*n*=3), G (*n*=6), G′ (*n*=4)], *Notch1* [C (*n*=6), C′ (*n*=3), H (*n*=6), H′ (*n*=6)], *Lef1* [D (*n*=4), D′(*n*=3), I (*n*=4), I′ (*n*=3)], and *Etv4* [E (*n*=6), E′ (*n*=3), J (*n*=6), J′ (*n*=4)] at E9.5 (A-E′) and E10.5 (F-J′). A-E and F-J show dorsal views of the posterior part of the body or tail in wild-type embryos. B′-E′ and F′-I′ show equivalent regions in *Sall4* cKO embryos. Black and blue arrows indicate normal expression in wild type (black) and reduced expression in *Sall4* cKO embryos, respectively. A-E′ and F-J′ are at the same scale.

Our previous study showed that *Msgn1*, a master regulator of PSM differentiation, was bound by SALL4 and was downregulated in *Sall4* cKO embryos (Tahara et al., 2019), suggesting the involvement of *Sall4* in regulation of PSM differentiation. Because *Sall4* is expressed in the entire PSM (Tahara et al., 2018), we followed up this observation and examined other genes expressed in the PSM. *Tbx6*, another crucial regulator of PM differentiation, was significantly downregulated in *Sall4* cKO embryos at E9.5 and was essentially undetectable at E10.5 (Fig. 2A,A′,F,F′). In ChIP-seq data, unlike *Msgn1*, we did not observe SALL4 enrichment at the *Tbx6* gene, suggesting that this regulation is indirect. Expression of *Delta1* (*Dll1*) and *Notch1*, which are crucial components of Notch signaling in the PSM, were downregulated but detectable in *Sall4* cKO embryos at E9.5 (Fig. 2B,B′,C,C′) and E10.5 (Fig. 2G,G′,H,H′). Among the members of the Notch family and the Delta family, SALL4 enrichment is observed around the TSS of the *Notch2* gene, introns of *Notch3* and *Notch4*, and 5′ upstream of *Dll1* (Table S1, Fig. S2). These results suggest that *Sall4* is directly and indirectly involved in regulation of gene expression in the PSM.

In the determination front model, a posterior-high to anterior-low gradient of WNT/β-catenin and FGF signaling is counteracted by an anterior-high to posterior-low gradient of retinoic acid signaling (Aulehla and Pourquié, 2010; Aulehla et al., 2008; Diez del Corral and Storey, 2004; Dubrulle et al., 2001; Sawada et al., 2001). We examined WNT/β-catenin and FGF signaling through the expression of genes regulated by these signaling pathways. *Lef1* is a mediator of and a target of WNT/β-catenin signaling and is strongly expressed in the PSM. *Lef1* expression in *Sall4* cKO embryos was reduced in comparison with wild-type embryos, and the expression domain along the anterior-posterior axis was shorter than wild type (Fig. 2D,D′,I,I′). *Etv4* is downstream of FGF signaling in the PSM. In addition to strong expression in the neural tube, *Etv4* is expressed in the posterior PSM (Fig. 2E,J). Compared with wild-type embryos, *Etv4* expression in *Sall4* cKO embryos was detected in smaller domains in the posterior PSM at E9.5 and E10.5 (Fig. 2E′,J′). These expression patterns suggest that WNT/β-catenin signaling and FGF signaling are present, but their levels are reduced in *Sall4* cKO PSM.

We also examined gene expression by qRT-PCR at E8.5, when *Sall4* recombination by *TCre* was detectable in the trunk tissue (Fig. S3A) (Akiyama et al., 2015). We observed downregulation of *Mesp2*, *Ripply2*, *Epha4* and *Efnb2* in *Sall4* cKO embryos, when compared with wild type (Fig. S3B-E). The degree of downregulation seems to be milder at E8.5 than E9.5 (Fig. 1B″-E″, Fig. S3B-E). This result suggests that levels of gene expression are reduced in the future thoracic levels at E8.5 in *Sall4* cKO, but the skeletal defects become evident at the lumbar level, where downregulation of gene expression is more significant than future thoracic levels. Altogether, these results indicate that expression of genes involved in PSM differentiation is impaired in *Sall4* cKO embryos and that the degree of reduction of gene expression correlates with posterior axial skeletal defects.

### *Sall4* regulates somite development

**Fig. 3.**
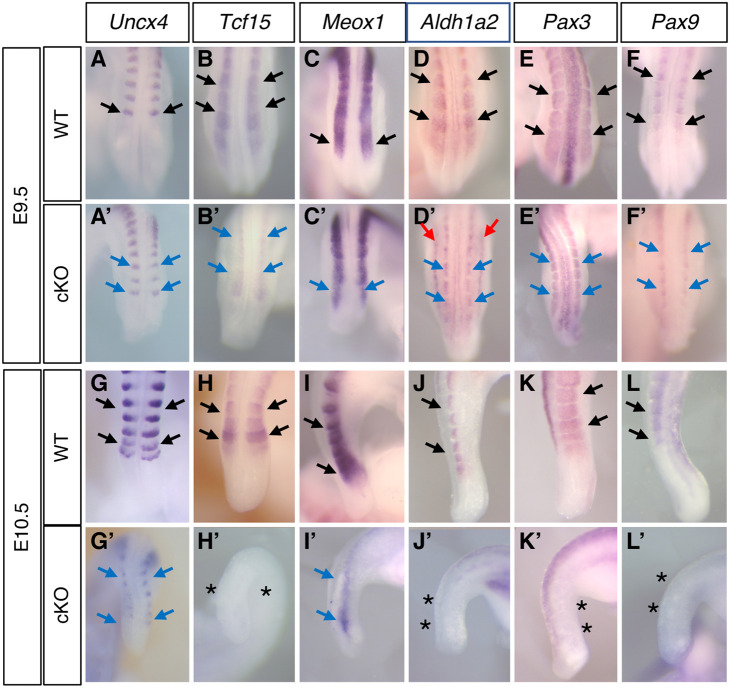
**Gene expression patterns in the** **somites.** (A-L′) Expression patterns of *Uncx4* [A (*n*=6), A′ (*n*=3), G (*n*=6), G′ (*n*=4)], *Tcf15* [B (*n*=6), B′ (*n*=5), H (*n*=6), H′ (*n*=4)], *Meox1* [C (*n*=4), C′ (*n*=4), I (*n*=4), I′ (*n*=3)], *Aldh1a2* [D (*n*=4), D′ (*n*=3), J (*n*=4), J′ (*n*=3)], *Pax3* [E (*n*=4), E′ (*n*=3), K (*n*=4), K′ (*n*=4)] and *Pax9* [F (*n*=4), F′ (*n*=3), L (*n*=4), L′ (*n*=3)] at E9.5 (A-F′) and E10.5 (G-L′). I-L and I′-L′ show lateral views; all other panels show dorsal views. Black and blue arrows indicate normal expression in wild type (black) and reduced expression in mutants, respectively. Red arrows in D′ indicate ectopic expression of *Aldh1a2* in *Sall4* cKO embryos. A-F′ and G-L′ are at the same scale. Asterisks indicate lack of expression.

Next, we examined the expression of genes involved in somite development. The expression domain of *Uncx4*, a marker of posterior somites, seemed narrower along the medio-lateral axis in *Sall4* cKO at E9.5 (Fig. 3A,A′). To clarify whether *Uncx4*-expressing domains are narrower in *Sall4* cKO, we measured the width of the most posterior *Uncx4*-expressing domain and found that the domain was indeed narrower in *Sall4* cKO than in wild type (Fig. S4A). The expression pattern of *Uncx4* further narrowed along the medio-lateral and anterior-posterior axes, and the signal became weak in *Sall4* cKO at E10.5 (Fig. 3G,G′). These expression patterns indicate severe reduction of the posterior trunk and tail size in *Sall4* cKO embryos.

*Tcf15* is required for anterior-posterior polarity of somites and its loss causes disorganized vertebrae formation (Burgess et al., 1996; Johnson et al., 2001). In *Sall4* cKO, *Tcf15* expression is downregulated at E9.5 (Fig. 3B,B′) and was undetectable at E10.5 (Fig. 3H,H′). *Meox1* is required for sclerotome polarity and axial skeleton formation (Mankoo et al., 2003; Skuntz et al., 2009). *Meox1* expression at E9.5 was not severely affected in *Sall4* cKO; however, its expression domain in the somite appears to be narrower than wild type along the medio-lateral axis (Fig. 3C,C′). At E10.5, *Meox1* expression in the tail region was severely downregulated in *Sall4* cKO embryos (Fig. 3I,I′). *Aldh1a2* is expressed in somites (Molotkova et al., 2005), and its expression in *Sall4* cKO embryos was restricted to the medial part of somites in *Sall4* cKO embryos at E9.5 (Fig. 3D,D′). At E10.5, *Aldh1a2* in the tail somites was undetectable (Fig. 3J,J′). Among these genes, SALL4 is enriched near the TSS of *Tcf15* and *Aldh1a2*, and 2.3 kb from the TSS of *Meox1* (Tables S1 and S2, Fig. S2), suggesting that SALL4 directly regulates these somite development genes. These results indicate that somites became smaller in *Sall4* cKO embryos at E9.5, and that normal somite gene expression is either lost or severely impaired at E10.5.

Consistent with the change in the somite morphology in *Sall4* cKO embryos, expression of dermomyotome marker *Pax3* and sclerotome marker *Pax9* was detected in a narrower region in *Sall4* cKO embryos at E9.5 (Fig. 3E-F′, Fig. S4B,C). Expression of both *Pax3* and *Pax9* was undetectable at E10.5 (Fig. 3K-L′). These analyses support the idea that, as in the PSM, *Sall4* regulates somite development, in part, by directly binding to those genes (Fig. S2).

### *Sall4* regulates trunk-tail transition and tail progenitors

The morphological defects of *Sall4* cKO mutants are clearly distinguishable posterior to the lumbar vertebrae, whereas cervical and thoracic levels are unaffected (Tahara et al., 2019). During body elongation, neuromesodermal progenitors are located in the posterior part of the body and contribute to development of the post-cranial body (Aires et al., 2018; Henrique et al., 2015; Steventon and Martinez Arias, 2017; Wilson et al., 2009). Later, the neuromesodermal progenitors relocate to the tail bud and contribute to tail elongation (Aires et al., 2018; Wilson et al., 2009). The defects in *Sall4* cKO mutants suggest that *Sall4* may have a role in the trunk-tail transition and/or maintenance of neuromesodermal progenitors in the tail bud. These processes rely on a network of *Gdf11*, *Lin28* and *Hox13* genes (Aires et al., 2019; Robinton et al., 2019). At E9.5, expression of *Gdf11* and *Lin28a* was downregulated in *Sall4* cKO, compared with wild-type embryos (Fig. S5A-B″), and their downregulation was more evident in the tail bud at E10.5 (Fig. S5C-D″). *Hoxb13* and *Hoxc13* are also downregulated in the tail bud at E10.5 (Fig. S5E-F″). These results support the idea that the trunk-tail transition and neuromesodermal progenitor activity in the tail are disrupted in *Sall4* cKO embryos, which may contribute to the defects in the posterior trunk and the tail.

### *Sall4* is required for accessible chromatin near a subset of genes

A recent study has shown chromatin accessibility analysis to be a useful strategy for investigating molecular and genetic mechanisms of somite development (Mok et al., 2021). To further understand how *Sall4* regulates PM development, we examined genome-wide chromatin accessibility via ATAC-seq. We used the mesoderm-enriched fraction of the posterior trunk at E9.5, similar to our previous ChIP-seq experiment (Tahara et al., 2019), from *Sall4* cKO and littermate *Sall4* conditional heterozygous (cHet) embryos as a control.

**Fig. 4.**
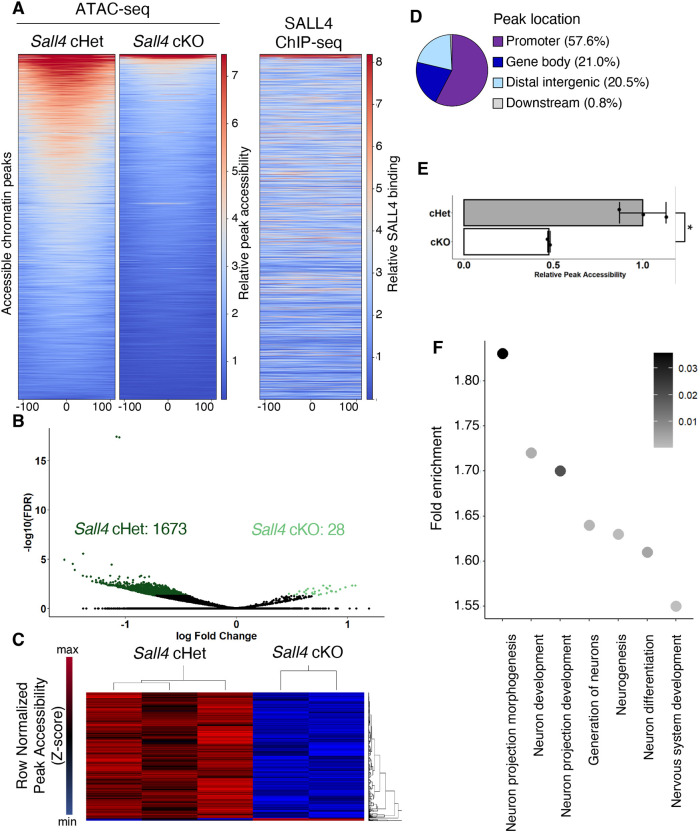
**Chromatin accessibility of mesoderm-enriched tissue from the posterior trunk.** (A) Heatmap showing genome-wide accessibility in *Sall4* cHet and *Sall4* cKO samples, and SALL4 enrichment in the same genomic intervals. The *x*-axis corresponds to ±100 base pairs from the center of accessible regions (shown as 0). (B) Volcano plot showing differentially accessible genomic intervals between *Sall4* cHet and *Sall4* cKO samples. (C) Heatmap of the 1701 genomic intervals that showed differential accessibility between *Sall4* cHet and *Sall4* cKO samples. (D) A pie chart showing distribution of differentially accessible regions. (E) A bar graph showing relative accessibility score between *Sall4* cHet and *Sall4* cKO samples. *P=0.0186 (Welch′s two-sample t-test). (F) A dot plot showing enriched GOs related to neural development and functions. Gray shading indicates FDR.

We found that 1673 genomic intervals are more accessible in cHet samples, whereas 28 sites are more accessible in cKO samples (Fig. 4A-C), indicating that *Sall4* acts to promote an open chromatin status in the trunk mesoderm. Analysis of the sequences of the differentially accessible regions showed that a large proportion of those regions are located in promoters (57.6% within 2 kb upstream of the TSS to 500 bp downstream of the TSS) or gene bodies (21%) (Fig. 4D, Table S3), suggesting that *Sall4* may regulate expression of these genes through changing accessibility for upstream transcription factors. Using deepTools, we scored the mean coverage density at the center of each differentially accessible region±100 bp as a measurement of accessibility (Ramírez et al., 2016). Relative accessibility was reduced ∼50% in *Sall4* cKO samples, when compared with cHet samples (Fig. 4E). Comparison of the differentially accessible regions with our SALL4 ChIP-seq data showed that the SALL4 enrichment does not correlate with the differentially accessible regions (Fig. 4A) (Tahara et al., 2019). These data suggest that the differential accessibility in the majority of 1701 sites is a downstream consequence of *Sall4*-dependent genetic programs. GO analysis of the differential accessible regions included enrichment of multiple GO terms related to neural differentiation (Fig. 4F, Table S4).

### *Sall4* enhances WNT signaling by regulating chromatin accessibility near positive and negative regulators of WNT signaling

WNT/β-catenin signaling and FGF signaling are major regulators of PSM development (Dequeant and Pourquié, 2008; Kageyama et al., 2012). GO analysis of RNA-seq data obtained from wild-type and *Sall4* cKO posterior trunk tissue (Tahara et al., 2019) showed that four WNT signaling-related GO terms are present in the top 100 GO terms (ranked at 3, 4, 5 and 73), whereas no GO terms related to FGF signaling appeared in the analysis (Table S5). Therefore, we examined the correlation between gene expression in the posterior trunk in the RNA-seq data and the current chromatin accessibility data of genes involved in WNT signaling.

**Fig. 5.**
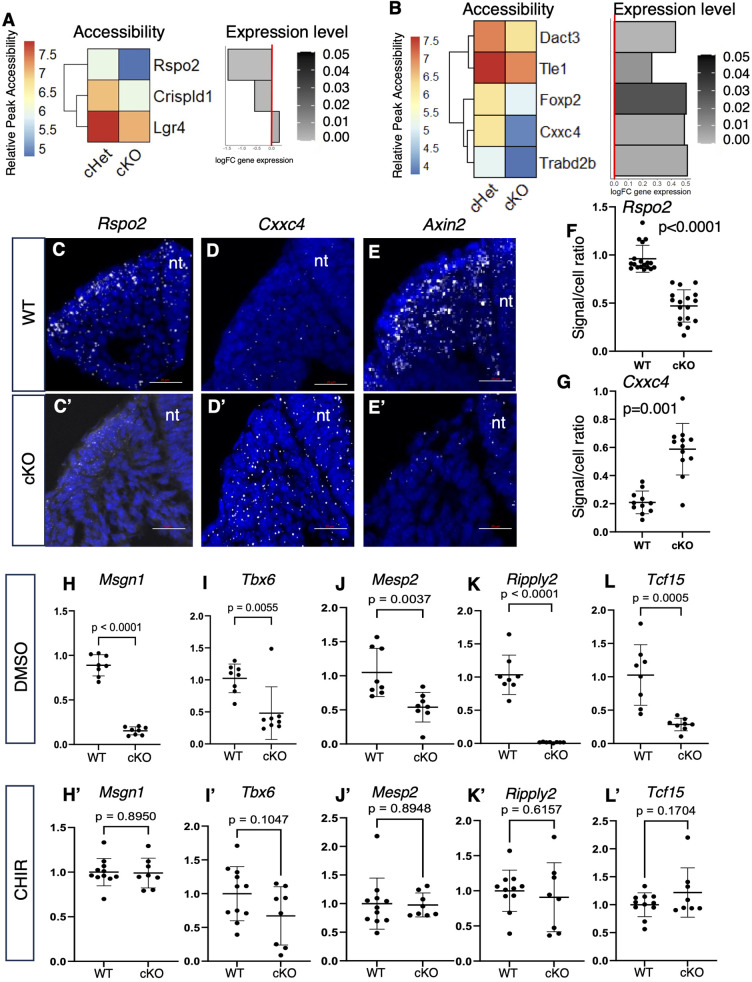
**WNT regulators with differential accessibility and differential expression between *Sall4* cHet and *Sall4* cKO.** (A) Heatmap and bar chart showing differential accessibility and expression of genes involved in activating WNT signaling. Gray shading indicates FDR. (B) Heatmap and bar chart showing differential accessibility and expression of genes involved in negative regulation of WNT signaling. Gray shading indicates FDR. (C-E′) RNA scope images of expression of *Rspo2* (C,C′), *Cxxc4* (D,D′) and *Axin2* (E,E′) in the PM of the posterior trunk in wild-type (C-E) and *Sall4* cKO (C′-E′) embryos at E9.5. DAPI signals and transcript signals are shown in blue and white, respectively. nt, neural tube. Scale bars: 20 µm. (F,G) Quantification of *Rspo2* (F) and *Cxxc4* (G) signals. Each dot represents data from a section. The *y*-axis shows the ratio between *Rspo2* or *Cxxc4* signal number and DAPI-positive nuclei number in the PM of the section. Data obtained from five wild-type and five *Sall4* cKO embryos. *P* values are indicated (unpaired *t*-test). (H-L′) Relative expression levels of *Msgn1* (H,H′), *Tbx6* (I,I′), *Mesp2* (J,J′), *Ripply2* (K,K′) and *Tcf15* (L,L′) in cultured E10.5 tail of wild-type or *Sall4* cKO embryos in the presence of DMSO (H-L) or CHIR (H′-L′). Each dot represents a cultured tail and *P* values are shown in each panel (unpaired *t*-test).

Given that *Sall4* primarily promotes an open chromatin status (Fig. 4), we searched for genes whose chromatin status became less accessible in *Sall4* cKO embryos. We found *Rspo2* (de Lau et al., 2011; Glinka et al., 2011), *Crispld1* (Khadjeh et al., 2020) and *Lgr4* (de Lau et al., 2011; Glinka et al., 2011), genes known to be positive regulators of WNT signaling, became less accessible in *Sall4* cKO (Fig. 5A). Among them, expression of *Rspo2* and *Crispld1* was downregulated in *Sall4* cKO embryos in our RNA-seq data (Fig. 5A). Indeed, downregulation of *Rspo2* expression was further supported by RNAscope (Fig. 5C,C′,F). This result suggests that SALL4 promotes expression of these WNT signaling activators by providing accessible TSS chromatin environment for upstream transcriptional activators.

We also found that the chromatin became less accessible in *Sall4* cKO embryos at sites near *Dact3* (Jiang et al., 2008), *Tle1* (Arce et al., 2009), *Foxp2* (Richter et al., 2021), *Cxxc4* (Hino et al., 2001) and *Trabd2b* (Zhang et al., 2012), which are known negative regulators of WNT signaling (Fig. 5B). Surprisingly, expression of these genes was upregulated in *Sall4* cKO embryos in the RNA-seq data (Fig. 5B). Among these genes, we further examined expression of *Cxxc4*, which is located distal to a region that exhibited the highest fold change in chromatin accessibility we detected. Gene expression analysis by RNAscope showed upregulation of *Cxxc4* in the PM (Fig. 5D,D′,G). These results indicate that SALL4 represses expression of these negative regulators of WNT signaling, and produces an accessible chromatin microenvironment at locations that may be of importance to transcriptional repressors.

Consistent with downregulation and upregulation of WNT signaling activators and repressors, respectively, expression of *Axin2*, which reports WNT/β-catenin signaling activity (Jho et al., 2002), was downregulated in the PM (Fig. 5E,E′). Collectively, these results suggest that *Sall4*-dependent chromatin accessibility contributes to promotion of WNT signaling.

In order to further examine whether regulation of WNT signaling by *Sall4* plays a role in gene expression in the PSM and somites, we sought to test whether activation of WNT signaling could rescue gene expression in *Sall4* cKO embryos. For this purpose, we dissected the tail from E10.5 embryos and set up *ex vivo* tail culture experiments, as previously performed by others (Fig. S6) (Bulusu et al., 2017). We compared responses to CHIR, which inhibits GSK3β and hence activates β-catenin signaling (Silva et al., 2008), at various culture durations. Expression of *Axin2* and *Lef1* was significantly elevated by CHIR treatment, compared with DMSO treatment, after 4 and 6 h (Fig. S6A,B), indicating that CHIR treatment for 4 h is sufficient to activate β-catenin signaling. With these data in mind, we cultured wild-type and *Sall4* cKO E10.5 tail with DMSO or CHIR for 4 h in order to examine whether activation of β-catenin signaling can rescue gene expression defects in the *Sall4* cKO tail (Fig. 5H-L′). Expression of *Msgn1*, *Tbx6*, *Mesp2*, *Ripply2* and *Tcf15* was downregulated in DMSO-treated *Sall4* cKO tails compared with DMSO-treated wild-type tails (Fig. 5H-L), consistent with downregulation of these genes in the *Sall4* cKO tail without culture (Figs 1, 2, 3) (Tahara et al., 2019). Treatment of cultured tail with CHIR showed that expression of these genes in *Sall4* cKO was comparable with that of wild-type tails (Fig. 5H′-L′). This result indicates that activating β-catenin signaling can rescue gene expression in the *Sall4* cKO tail and supports our notion that β-catenin signaling acts downstream of *Sall4*.

### *Sall4* negatively regulates neural genes in the posterior trunk mesoderm

**Fig. 6.**
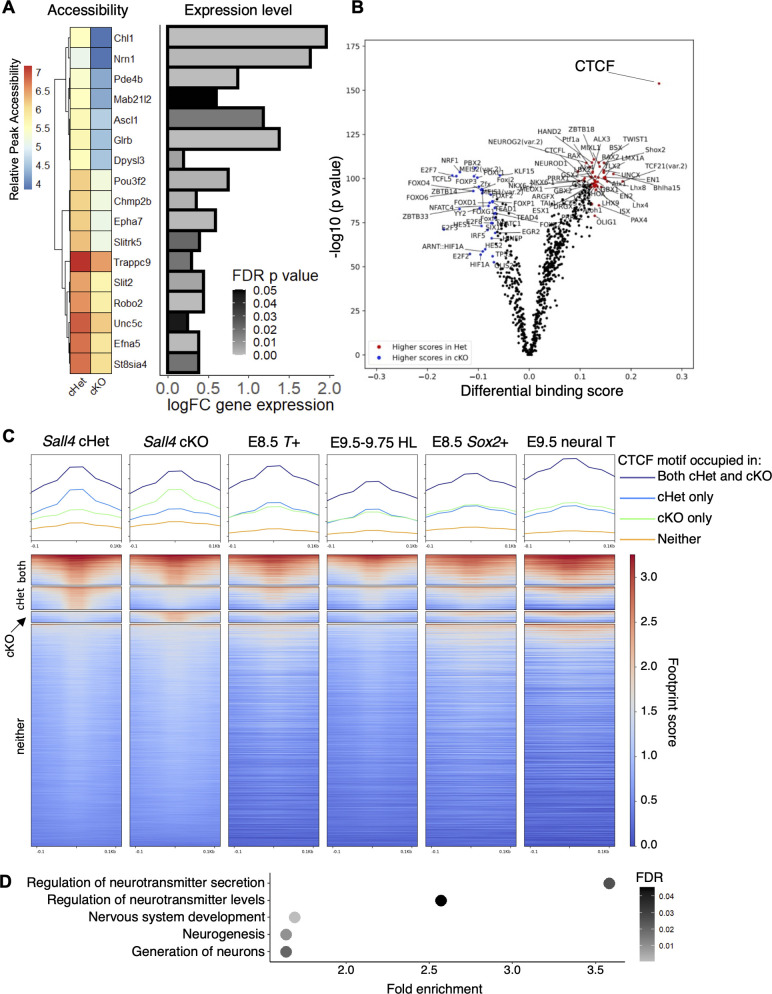
***Sall4* represses neural gene expression in mesoderm through regulation of chromatin accessibility.** (A) Heatmap and bar chart showing differential accessibility and expression of genes related to neural differentiation. (B) Volcano plot of footprinting of differential accessible regions. The data point for CTCF is indicated. (C) Heatmap of inferred transcription factor occupancy at 11,443 occurrences of CTCF motifs in *Sall4* cHet and *Sall4* cKO samples. The heatmap shows, from the top to bottom, motifs occupied in both cHet and cKO, followed by motifs significantly more occupied in cHet, motifs significantly more occupied in cKO and motifs not occupied in either cHet or cKO. The *x*-axis corresponds to ±100 base pairs from the center of accessible regions (shown as 0). Heatmaps of the CTCF motifs of E8.5 *T*-expressing cells, E9.5-E9.75 nascent hindlimb bud cells (HL), E8.5 *Sox2*-expressing cells and E9.5 neural tube cells are also shown. (D) Dot plot showing enrichment of neural-related GO terms, obtained from a list of genes closest to the CTCF motifs preferentially bound in *Sall4* cHet embryos.

GO analysis of differentially accessible regions showed that GO terms involving neurogenesis and neuronal development were over-represented (Fig. 4F, Table S4). Interestingly, our previous analysis showed that expression of mesodermal and neural genes was downregulated and upregulated, respectively, in *Sall4* cKO embryos (Tahara et al., 2019). Therefore, we compared differentially expressed mesodermal and neural genes with the chromatin accessibility data. Upon examination of the nearest genes to the 1701 differentially accessible regions, we did not find genes currently annotated as related to mesoderm development. However, we did find that 17 sequences are located close to genes whose functions are associated with neural lineage development, such as neurogenesis factor *Ascl1* (Castro et al., 2011) (Fig. 6A). These genes are upregulated in *Sall4* cKO embryos, and all 17 nearby differentially accessible regions are less accessible in *Sall4* cKO embryos. These correlations suggest that *Sall4* promotes accessibility of chromatin near these neural genes that contributes to repression of their expression.

Having characterized differential accessibility in the presence or absence of *Sall4*, we next wished to determine the activity of which DNA binding factor may be impacted by the altered chromatin landscape. To do this, we performed a genome-wide footprinting analysis with TOBIAS (Bentsen et al., 2020), which can detect evidence of factor binding by characterizing small areas of poor read coverage flanked by relatively accessible regions (Louise Smith et al., 2022; Sung et al., 2016). This analysis showed that some transcription factor-binding motifs exhibited higher occupancy in cHet or cKO samples, indicated by differential binding scores (Fig. 6B). Among all the motifs, the CTCF binding motif exhibited the highest score in cHet samples (Fig. 6B, Table S6), indicating that CTCF binding was strongly enriched in the presence of *Sall4.*

Although most of the 11,443 CTCF binding motifs in the genome were not occupied in both *Sall4* cKO and *Sall4* cHet samples, we detected 1570 sites occupied in both *Sall4* cKO and *Sall4* cHet samples (Fig. 6C, Table S6). We detected 1182 sites occupied in cHet but not cKO, and 569 sites occupied in cKO but not in cHet (Fig. 6C). To determine whether the observed occupancy of CTCF sites is specific to the posterior trunk mesoderm, we performed footprinting analysis of publicly available ATAC-seq data and compared them with our data. We found that CTCF occupancy in mouse embryonic stem cells (Metzis et al., 2018; Tafessu et al., 2023) and mouse embryonic fibroblasts (Wang et al., 2019) exhibited significantly different CTCF occupancy patterns when compared with posterior trunk mesoderm (Fig. S7). Therefore, we next compared our data with cells/tissues obtained from mouse embryos. *T*-expressing mesoderm progenitors from E8.5 embryos (Koch et al., 2017) and nascent hindlimb buds from E9.5-9.75 embryos, which are derived from the lateral plate mesoderm (Koyano-Nakagawa et al., 2022), shared some sites with detected CTCF occupancy in the posterior trunk mesoderm (Fig. 6C). However, we did not detect a significant correlation between CTCF binding in mesoderm progenitors/nascent hindlimb buds and that of the posterior trunk mesoderm (Fig. 6C). Specifically, areas with the strongest detected CTCF binding signal either only in *Sall4* cHet or *Sall4* cKO in the posterior trunk mesoderm appeared to show limited CTCF binding activity in mesoderm progenitors and nascent hindlimb buds. We also compared our data with *Sox2*-expressing neural progenitors from E8.5 embryos and E9.5 neural tube (Koch et al., 2017; Metzis et al., 2018), which also did not exhibit significant correlation to posterior trunk mesoderm (Fig. 6C). Some CTCF occupied sites are shared in E8.5 *T*^+^ cells, E8.5 *Sox2*^+^ cells, E9.5-9.75 hindlimb bud mesoderm, E9.5 neural tube and in the posterior trunk. These CTCF occupied areas might function commonly in different cell types for fundamental cellular activities. These results suggest that differential occupancy of CTCF binding sites between cHet and cKO is specific to the posterior trunk mesoderm. CTCF is crucial for proper regulation of gene expression and embryonic development, and CTCF is known to repress gene expression (Herold et al., 2012; Kim et al., 2015). Given our observation that *Sall4*-dependent chromatin accessibility is paired with reduced expression of nearby genes (Fig. 6A), we investigated the fraction of CTCF footprints differentially detected in *Sall4* cHet and cKO. Gene ontology analysis of genes closest to these differentially occupied CTCF sites shows enrichment of neural differentiation genes (Fig. 6D, Table S7). These results and the well-characterized repressive function of CTCF collectively suggest that *Sall4* contributes to PM development in the posterior trunk by repressing neural gene expression through regulation of CTCF insulator sequence binding.

## DISCUSSION

**Fig. 7.**
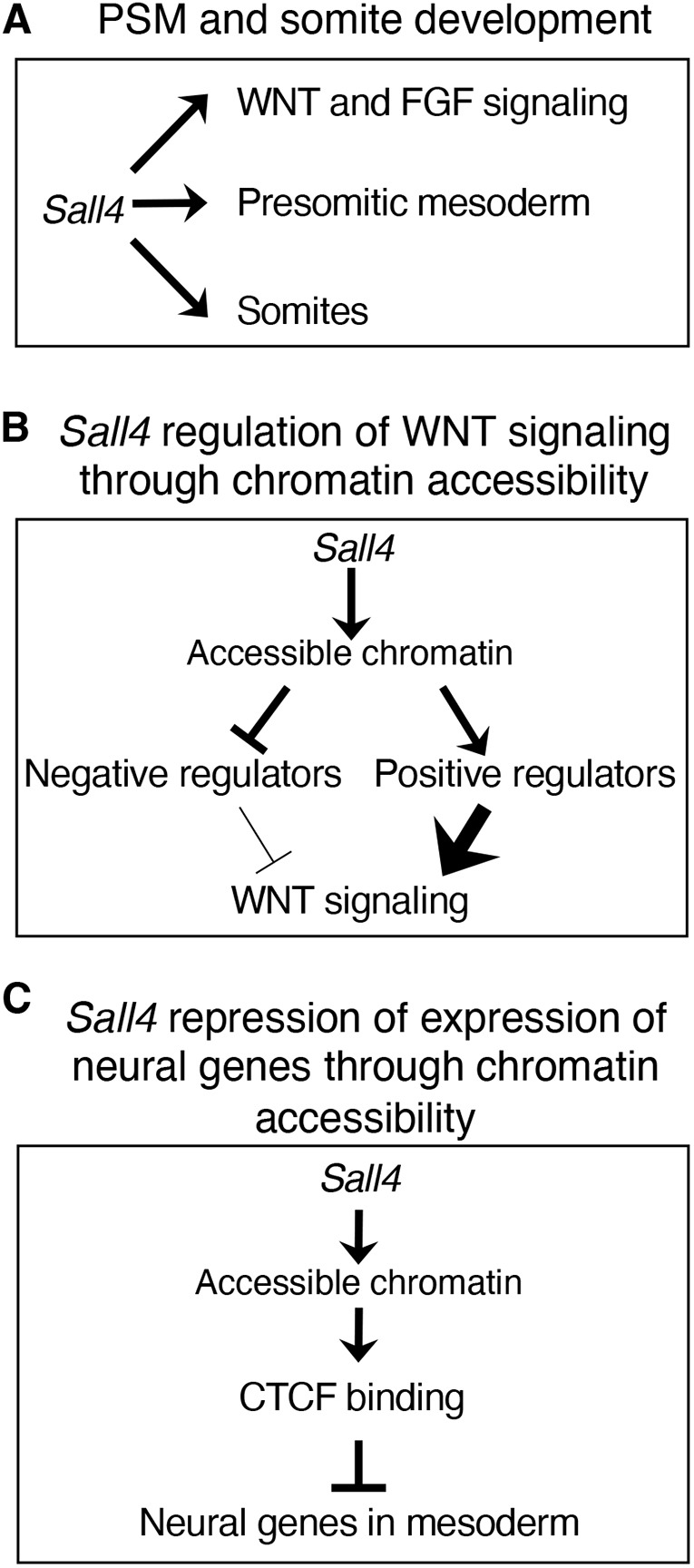
**Models for *Sall4* functions in PM development.** (A) *Sall4* contributes to multiple aspects of PSM development and somite development. (B) *Sall4*-dependent open chromatin status drives repression and activation of negative regulators and positive regulators, respectively, of WNT signaling. (C) *Sall4*-dependent open chromatin status contributes to repression of neural gene expression in the mesoderm, by facilitating CTCF binding.

In this study, we have investigated mechanisms by which *Sall4* regulates PM development using genetic and genomic approaches. *In situ* hybridization experiments, coupled with RNA-seq and ChIP-seq data analysis, suggest that *Sall4* has a variety of roles in PSM development. In the PSM, *Sall4* contributes to regulation of WNT and FGF signaling, and helps to maintain the expression of genes involved in Notch signaling and somite boundary formation (Fig. 7A). *Sall4* also promotes somite development (Fig. 7A). Furthermore, ATAC-seq analysis demonstrates that *Sall4*-dependent chromatin accessibility regulates activation and repression of a subset of genes encoding WNT signaling activators and repressors, respectively (Fig. 7B). *Sall4*-dependent chromatin accessibility also facilitates repression of neural differentiation gene expression in the PSM (Fig. 7C). These mechanisms act together to regulate posterior trunk development in the developing embryo.

### Role of *Sall4* in PM development

Deleting *Sall4* by *TCre* caused severe axial skeleton defects in the posterior trunk and the tail. Analysis of the gene expression pattern showed defects in multiple genes that are expressed in specific developmental processes. Those include genes expressed in the PSM, during somitogenesis and during somite differentiation. The reduced expression of those genes suggests that *Sall4* is broadly involved in PM development. In addition, by re-visiting our recent SALL4 ChIP-seq data, we found that several, but not all, analyzed genes were bound by SALL4 around the TSSs. This suggests that *Sall4* directly and indirectly participates in regulation of PM development.

Gene expression analysis in *Sall4* cKO embryos demonstrated that the defects were relatively mild at E9.5 and became more severe at E10.5. Several mechanisms could explain this observation. The timing of E9.5 to E10.5 correlates with the transition of trunk to tail development. A simple interpretation is that *Sall4* has distinct roles in the development of the trunk and tail, which possess distinct developmental programs (Aires et al., 2019; Jurberg et al., 2013; Robinton et al., 2019). *Sall4* function may be more significant in tail development than in posterior trunk development, as observed by downregulation of the *Gdf11-Lin28-Hox13* system. A second interpretation is that *Sall4* acts on axial skeleton development in a broad region, and the observed differences in the degree of gene expression defects reflect the timing of SALL4 protein depletion. Our previous analysis of SALL4 protein expression in *Sall4* cKO embryos showed that SALL4 protein was depleted at E9.5-E10.5 in the posterior part of the body (Tahara et al., 2019), although *TCre*-mediated recombination occurs as early as E7.5 (Perantoni et al., 2005). The observed timing of the transition to severe defects correlates with this timing of depletion of SALL4, which suggests that milder defects at E9.5 may be due to residual SALL4 protein at this stage. A third mechanism involves possible redundancy with other members of the Sall gene family. For example, our previous study demonstrated that *Sall1* and *Sall3* redundantly regulate autopod development in mice (Kawakami et al., 2009). A *Sall2* knockout; *Sall4* gene trap allele exhibits increased neural tube closure defects when compared with *Sall2* knockout in mouse embryos (Böhm et al., 2008), which also suggests functional redundancy among Sall genes. Moreover, *Sall4*^+/−^; *Sall1*^+/−^ mice exhibit renal agenesis and anal stenosis, demonstrating genetic interactions between Sall genes (Sakaki-Yumoto et al., 2006). Additionally, *Sall4*^−/−^; *Sall1*^−/−^ mouse embryonic stem cells exhibit more significant spontaneous upregulation of neural gene expression, compared with *Sall4*^−/−^ or *Sall1*^−/−^ embryonic stem cells (Miller et al., 2016). It is possible that other Sall members may partially compensate for the loss of *Sall4*, which might contribute to relatively mild defects at E9.5. These scenarios are not mutually exclusive; it is possible two or all the three scenarios simultaneously act in developing embryos.

### *Sall4*-dependent chromatin accessibility regulates positive and negative regulators of WNT signaling

In addition to the analysis of RNA-seq and ChIP-seq data, our ATAC-seq results suggested additional mechanisms by which *Sall4* regulates PM development through regulators of WNT signaling. High levels of WNT signaling in PSM maintains cells in an undifferentiated state (Aulehla and Pourquié, 2010). Our recent study of *Sall4* cKO embryos demonstrated that *Sall4* is required for promoting WNT signaling in neuromesodermal progenitors (Tahara et al., 2019). A reduced expression domain of *Lef1* and downregulation of *Axin2*, which are targets of WNT signaling (Filali et al., 2002; Jho et al., 2002), suggest that WNT signaling is reduced in the PSM in *Sall4* cKO embryos. Rescued expression levels of several genes expressed in the PSM or somites in the *Sall4* cKO tail after CHIR treatment also supports the notion that β-catenin signaling acts downstream of *Sall4* to regulate expression of PSM and/or somite genes.

Our previous analysis of neuromesodermal progenitors demonstrated downregulation of WNT signaling in *Sall4* cKO embryos; however, how *Sall4* promotes WNT signaling remained unknown. The analysis of chromatin accessibility in the posterior trunk mesoderm in *Sall4* cHet and cKO led us to discover a correlation between upregulation of gene expression and less accessible chromatin status in several negative regulators of WNT signaling. Our observation suggests that *Sall4*-dependent chromatin accessibility contributes to an open chromatin status that would allow repressor complex access and negatively regulate these negative regulators of WNT signaling. Similarly, our data also suggest that *Sall4*-dependent chromatin accessibility contributes to activator complex access and upregulates some positive regulators of WNT signaling. WNT signaling is a crucial regulator of development of a variety of tissues and organs, and its level must be tightly regulated by positive and negative regulators (Clevers and Nusse, 2012; Steinhart and Angers, 2018). *Sall4*-dependent regulation of a set of regulators of WNT signaling may also operate in tissues with high levels of *Sall4* expression.

### Negative regulation of the neural program in the mesoderm tissue

Similar to WNT signaling, our data support the notion that *Sall4* regulates a set of neural genes by creating an open chromatin microenvironment for upstream repressive factors in the posterior trunk mesoderm. Our ATAC-seq analysis uncovered an association between downregulated neural gene expression and CTCF-binding footprints near several neural differentiation genes in the posterior trunk mesoderm. Like the negative regulators of WNT signaling, *Sall4* may repress neural genes by making regions of importance accessible to repressor proteins. Interestingly, *Sall4* repression of neural gene expression has been also observed in other systems. Specifically, we have reported that *Sall4* cKO by *TCre* caused expanded neural tissue at the expense of mesoderm development from neuromesodermal progenitors (Tahara et al., 2019). The same study also demonstrated an accelerated neural differentiation program evidenced by early expression of motor neuron marker ISL1 in the neural tube in *Sall4* cKO embryos, when compared with wild-type embryos. Moreover, loss of *Sall4* in mouse embryonic stem cells caused spontaneous upregulation of neural genes, whereas expression of pluripotency-related genes is maintained (Miller et al., 2016). In the study of mouse embryonic stem cells, it has been shown that SALL4 binds to putative enhancer sequences and blocks expression of differentiation-promoting genes (Miller et al., 2016; Ru et al., 2022). These reports and our current study indicate that restricting the neural program seems to be a common function of *Sall4*.

*Sall4* is broadly and highly expressed in the neural tube (Kohlhase et al., 2002; Tahara et al., 2018); however, neural genes are expressed in the neural tube. This observation raises a question: does *Sall4* have a role in neural cells? Our previous study demonstrated that *Sall4* cKO in neuromesodermal progenitors by *TCre* caused accelerated neural differentiation in the neural tube in the posterior trunk (Tahara et al., 2019). This observation indicates that SALL4 also represses neural program in the neural tube, but it does not completely suppress high levels of neural gene network when compared with the mesoderm tissue. Such repression in neural cells may involve SALL4-dependent chromatin accessibility, as we observed in the mesoderm tissue in this study. It is also possible that SALL4 function may require cell type-specific molecular partners. For example, while SALL4 directly binds to AT-rich sequences in mouse embryonic stem cells (Pantier et al., 2021; Ru et al., 2022), SALL4 indirectly binds to DNA via its interaction partner PLZF in spermatogonial stem cells (Lovelace et al., 2016). The presence or absence of SALL4 molecular partners may explain the different degrees of repression of neural gene expression in mesodermal versus neural cells.

We highlighted differential CTCF binding of potential insulators near neural genes as one mechanism by which this regulation occurs. Footprinting analysis of ATAC-seq data indicated the highest score for CTCF motifs in the *Sall4*-dependent differentially accessible regions. Given that a major role of CTCF is regulating gene expression, the reduced CTCF score in *Sall4* cKO embryos would be expected to change gene expression in *Sall4* cKO embryos. This mechanism may contribute to changes of global gene expression in *Sall4* cKO embryos, including neural genes in the posterior trunk mesoderm. These reports and our current study support the idea that *Sall4* functions to negatively regulate neural development.

## MATERIALS AND METHODS

### Animal breeding and *in situ* hybridization

Embryos were collected by timed mating of *Sall4^fl/fl^* females and *TCre^Tg/Tg^; Sall4^+/fl^* males (Akiyama et al., 2015). Animal breeding was performed according to the approval by the Institutional Animal Care and Use Committee of the University of Minnesota. After fixation in 4% paraformaldehyde in PBS-Tween 20 overnight, embryos were washed and dehydrated in graded series of PBT/methanol and stored in 100% methanol. Three to six embryos per probe per stage were examined by whole-mount *in situ* hybridization.

### RNAscope

*In situ* hybridization was carried out with a RNAscope Multiplex Fluorescent Reagent Kit v2 (Advanced Cell Diagnostics). Embryos were fixed in 4% paraformaldehyde overnight at 4°C and cryosectioned at 14 µm. After the sections were dried, OCT compound around the sections was removed with 1×PBS for 3 min, followed by dehydration in an ascending ethanol series (50%, 70%, 100% and 100%). After applying hydrophobic pen around sections, slides were placed in 3% hydrogen peroxide in methanol for 15 min, followed by washes in water for 3 min. After this step, we used the manufacturer's protocol for fresh frozen sections, including the step with Protease IV at room temperature for 30 min. Hybridization was carried out with the mouse *Axin2* probe (400331), mouse *Cxxc4* probe (1184501-C2) and mouse *Rspo2* probe (402001-C2 (all from Advanced Cell Diagnostics). Detection of the probes was performed with Opal dyes 520 (*Axin2*), 650 (*Cxxc4*) and 570 (*Rspo2*). Fluorescent images were acquired with a Zeiss LSM710 with Zen software.

### Quantitative PCR analysis

E9.5 embryos were cut at the somite-PSM boundary using a carbon steel scalpel (10316-14, Fine Science Tools) and the posterior tissue was collected. E10.5 embryos were cut at the 4th and 5th somite boundary for collecting the tail tissue. E8.5 embryos were cut at the anterior edge of the first somite for collecting the trunk tissue. Total RNA was isolated using the Direct-zol RNA MicroPrep kit (Zymo Research) and cDNA was synthesized using iScript cDNA synthesis kit (BioRad) according to the manufacturers' instructions. qPCR was performed using PowerUp SYBR Green Master Mix (A25742, ThermoFisher) and primers in Table S8. Gene of interest expression was normalized to expression of the housekeeping gene *Actb* (β-actin).

### Tail *ex vivo* culture

Mouse embryos were dissected in PBS with 1% FBS. The tail was cut at the 4th and 5th somite boundary from E10.5 embryos, and culture experiments were carried out according to a published procedure (Bulusu et al., 2017). The tails were placed on Transwell (3470, Corning), and cultured at the liquid-air interface. The culture medium was DMEM/F12, supplemented with 20% (v/v) rat serum and DMSO or CHIR99021 (10 µM, R&D Systems). The culture plate was incubated in a humidified chamber at 37°C in 5% CO_2_, 60% O_2_ and 35% N_2_.

### ATAC-seq

Embryos at E9.5-9.75 were collected by timed mating of *Sall4^fl/fl^* females and *TCre^Tg/Tg^; Sall4^+/fl^* males, and *Sall4* cKO embryos were identified by short and thin posterior trunks. *Sall4* cHet and cKO embryos were separately pooled and processed. Each embryo was genotyped by genomic PCR to confirm the genotype after the ATAC reaction. Tissues posterior to the boundary of PSM and the somite were collected and kept in PBS on ice during dissection. The dissected tissues were treated by dispase (1.5 mg/ml, Roche, 4942078001) at 37°C for 5 min, followed by removal of the neural tube using a tungsten needle in ice-cold PBS, as previously described (Tahara et al., 2019). The remaining tissue (the posterior mesoderm tissue) was dissociated by TrypLE (Invitrogen) at 37°C for 5 min, neutralized by DMEM+10% FBS, and collected by low-speed centrifugation (500 ***g*** for 3 min at room temperature). The ATAC reaction was performed as described previously (Buenrostro et al., 2015), and libraries were created using dual indexed Nextera reagent kits. The sequencing was performed by NextSeq Midoutput 2×75 bp Run and generated 18.4 M mean read-pairs per sample.

### ATAC-seq data processing and analysis

Reads were trimmed using TrimGalore (0.6.0) (https://doi.org/10.5281/zenodo.5127899) and mapped to the GRCm38 genome using bwa mem (v 0.7.17) (https://doi.org/10.48550/arXiv.1303.3997). Mapped reads were sorted with samtools (v1.9) (Danecek et al., 2021) and duplicates were marked with Picard MarkDuplicates (2.18.16; https://broadinstitute.github.io/picard/). ATAC peaks were identified with MACS (v2.1.1.20160309; https://doi.org/10.5281/zenodo.3748809) using the following command line options: callpeak –broad -q 0.05 –nomodel –shift -100 –extsize 20.

Differential accessibility testing was performed with DiffBind using default parameters (Ross-Innes et al., 2012; Stark and Brown, 2011). Differentially accessible peaks were annotated using default parameters in ChIPpeakAnno (Zhu et al., 2010) with Ensembl annotation data from release 79 (Cunningham et al., 2022). Local chromatin accessibility was scored using the computeMatrix function in deepTools2 (Ramírez et al., 2016) via Galaxy (Afgan et al., 2018) on genomic regions of interest. Footprinting analysis was caried out with the TOBIAS snakemake pipeline (Bentsen et al., 2020) using transcription factor binding motifs from the JASPAR database (Fornes et al., 2020). GO analysis was performed using PANTHER (Mi et al., 2019; Thomas et al., 2022). The BioProject accession number of ATAC-seq in the GEO database is GSE217671.

### ChIP-seq data processing and analysis

Sequencing data was obtained from the Sequence Read Archive database (BioProject accession number PRJNA525663). Reads were mapped to the mm10 genome with Bowtie2 (Langmead and Salzberg, 2012). Potential PCR duplicates were removed with Samtools rmdup (Danecek et al., 2021). Peaks were called on replicates with Macs2 (Jeon et al., 2020) and differential binding testing was carried out with DiffBind (Ross-Innes et al., 2012).

### RNA-seq data processing and analysis

Sequencing data were obtained from the Sequence Read Archive database (BioProject accession number PRJNA525663). Analysis used the previously published CHURP pipeline (https://doi.org/10.1145/3332186.3333156). Briefly, quality control was carried out with fastQC (https://www.bioinformatics.babraham.ac.uk/projects/fastqc/), and reads were trimmed with Trimmomatic (Bolger et al., 2014). Hisat2 (Kim et al., 2019) was used to align reads to the GRCm38 genome. Aligned reads were sorted with SAMtools (Danecek et al., 2021) and gene counts were determined with featureCounts (Liao et al., 2014). Differential expression testing with edgeR revealed differentially expressed genes (Robinson et al., 2010).

## Supplementary Material



10.1242/develop.202649_sup1 Supplementary information

Table S1. Genomic regions with enriched SALL4 binding in the posterior trunk mesoderm

Table S2. Regions with enriched SALL4 binding in and around select genes related to this study

Table S3. Differentially accessible regions between *Sall4* cHet and *Sall4* cKO posterior trunk mesoderm

Table S4. Results of gene ontology analysis of genes nearest to differentially accessible regions between *Sall4* cHet and *Sall4* cKO posterior trunk mesoderm

Table S5. Results of gene ontology analysis of differentially expressed genes between WT and *Sall4* cKO posterior trunk tissue

Table S6. CTCF occupancy scores of CTCF binding motifs in *Sall4* cKO and *Sall4* cHet samples from footprinting analysis of ATAC-seq data

Table S7. Results of gene ontology analysis of genes closest to preferentially occupied CTCF sites in *Sall4* cHet relative to *Sall4* cKO posterior trunk mesoderm
