# Supplementary material for: Sall4 regulates posterior trunk mesoderm development by promoting mesodermal gene expression and repressing neural genes in the mesoderm
Source: Development. 2024 Mar 4;151(5):dev202649. doi: 10.1242/dev.202649 (PMC10946440; doi:10.1242/dev.202649)
Supplement: Supplementary information [file develop-151-202649-s1.pdf]

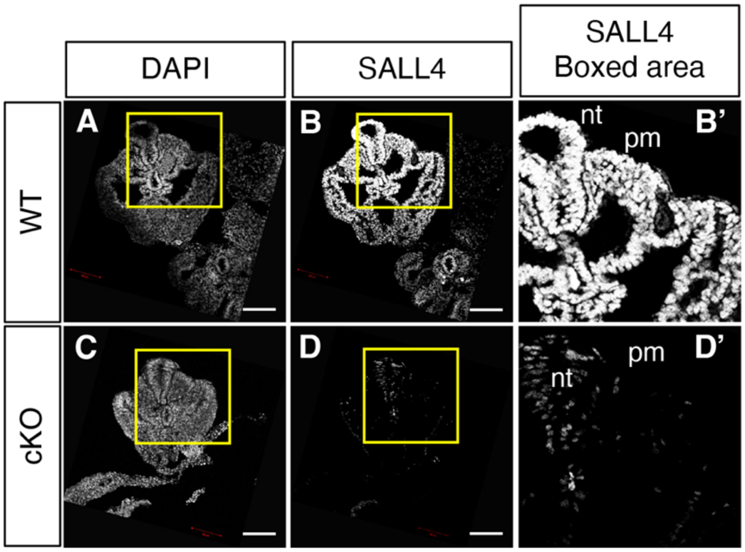

Fig. S1. SALL4 immunofluorescence on WT and *Sall4* cKO embryos

Confocal images after SALL4 immunofluorescence on cross sections of the posterior trunk of WT (A-B', n=17) and *Sall4* cKO (C-D', n=23) embryos at E9.5. DAPI signals (A, C) and SALL4 signals (B, B', D, D') are shown in a black/white mode. B' and D' show the boxed areas in B and D, respectively. With the settings to detect residual SALL4 immunoreactivity in *Sall4* cKO sections, the SALL4 signals in the WT embryos were essentially saturated. nt: neural tube, pm: paraxial mesoderm. Scale bar, 100 µm.

Development • Supplementary information

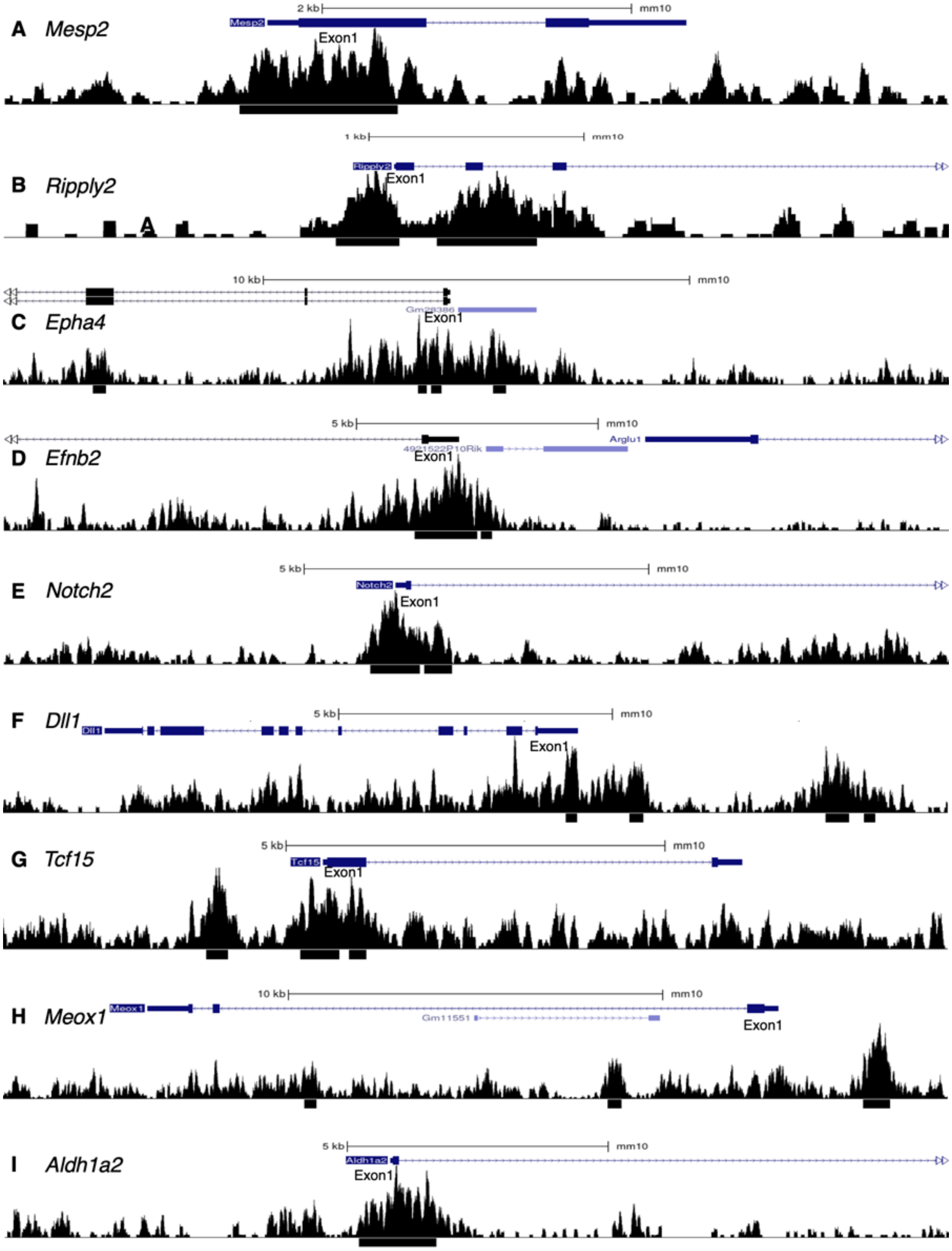

Fig. S2. SALL4 ChIP-seq tracks of genes expressed in PM

SALL4 ChIP-seq tracks of *Mesp2* (A), *Ripply2* (B), *Epha4* (C), *Efnb2* (D), *Notch2* (E), *Dll1* (F), *Tcf15* (G), *Meox1* (H) and *Aldh1a2* (I). Black bars under the track indicate statistically significant SALL4 enrichment.

Development • Supplementary information

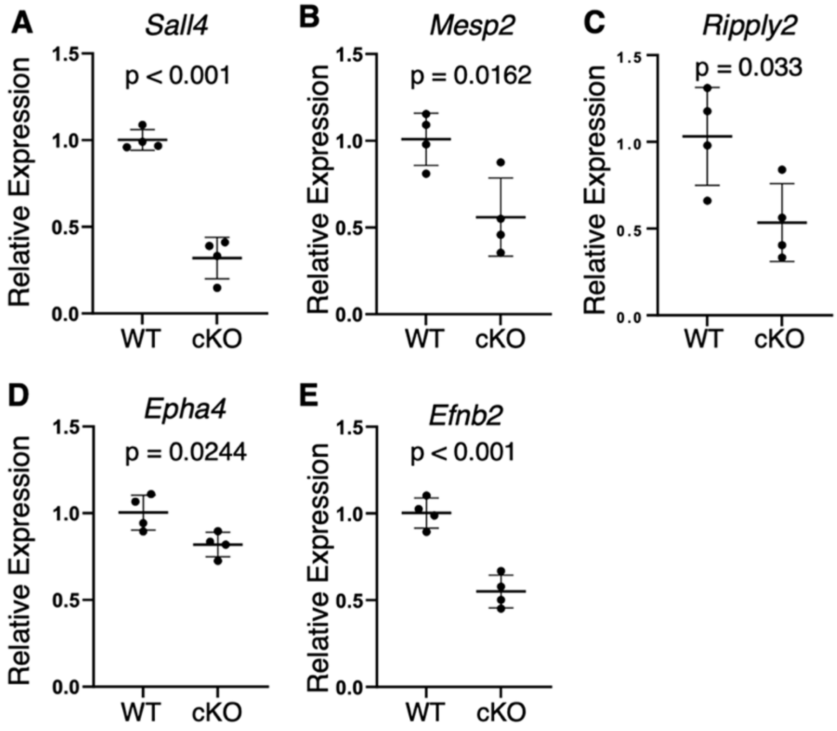

Fig. S3. Gene expression in E8.5 trunk between WT and *Sall4* cKO

Relative expression of *Sall4* (A), *Mesp2* (B), *Ripply2* (C), *Epha4* (D) and *Efnb2* (F) in the trunk of E8.5 WT and *Sall4* cKO embryos.

Each dot represents an embryo. Eight to ten somite stage embryos were used. P values by unpaired t-test are shown in each panel.

Development • Supplementary information

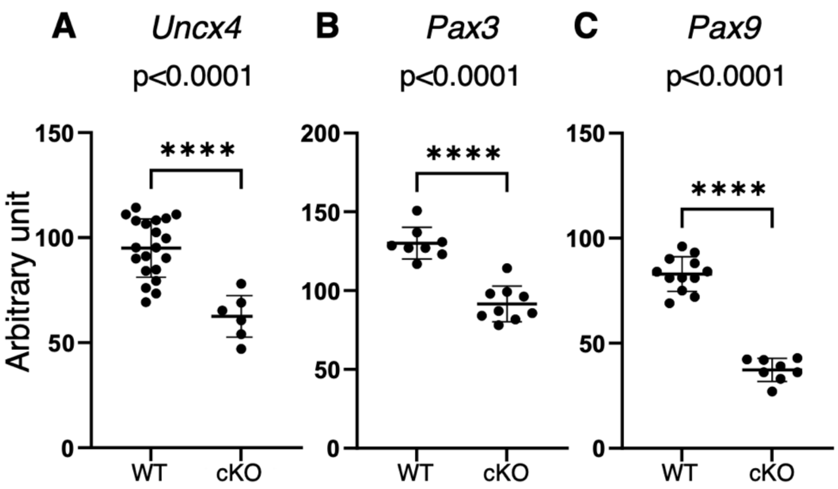

Fig. S4. Measurement of the width of the most posterior expression domains of *Uncx4* (A), *Pax3* (B), and *Pax9* (C) in WT and *Sall4* cKO embryos at E9.5

Dorsal viewed images were imported into FIJI, and the width of the most posterior expression domain was measured. Each dot represents a somite, and p values by unpaired t-test are shown in each panel.

Development • Supplementary information

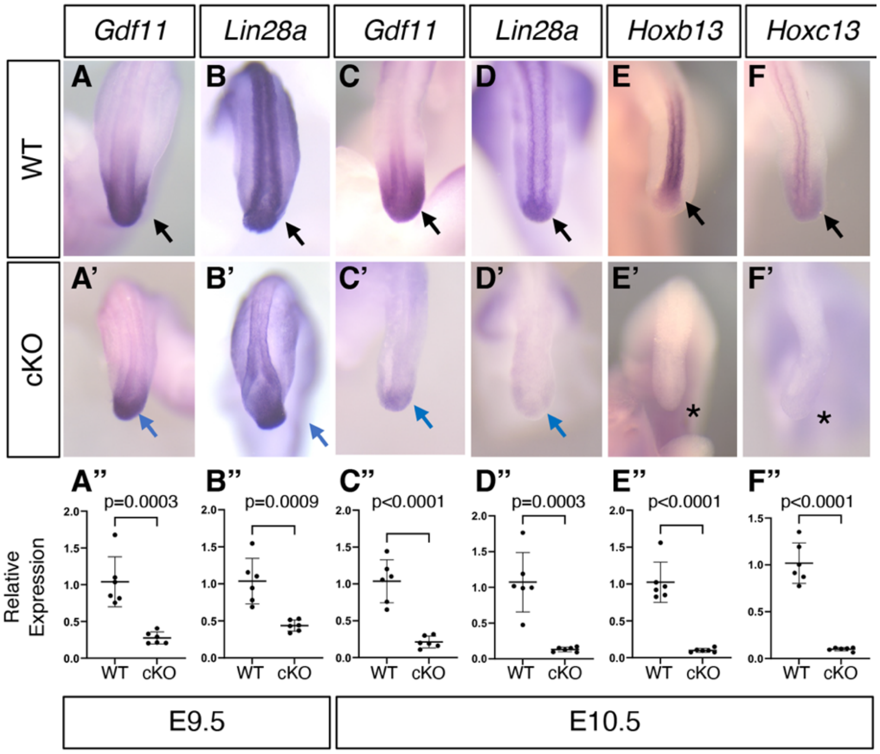

Fig. S5. Analysis of gene expression involved in trunk-tail transition

(A- F") Dorsal views of the posterior part of WT and *Sall4* cKO embryos hybridized with *Gdf11* (A, A', C, C'), *Lin28a* (B, B', D, D'), *Hoxb13* (E, E') and *Hoxc13* (F, F') probes. E9.5 (A-B') and E10.5 (C-F') embryos are shown. Black arrows and blue arrows point to normal and reduced expression, respectively. Asterisks indicate lack of expression.

(A"-F") Relative expression levels of *Gdf11* (A", C"), *Lin28a* (B", D"), *Hoxb13* (E") and *Hoxc13* (F") at E9.5 (A", B") and E10.5 (C"-F") are shown. Each dot represents an embryo, and p values by unpaired t-test are shown in each panel.

Development • Supplementary information

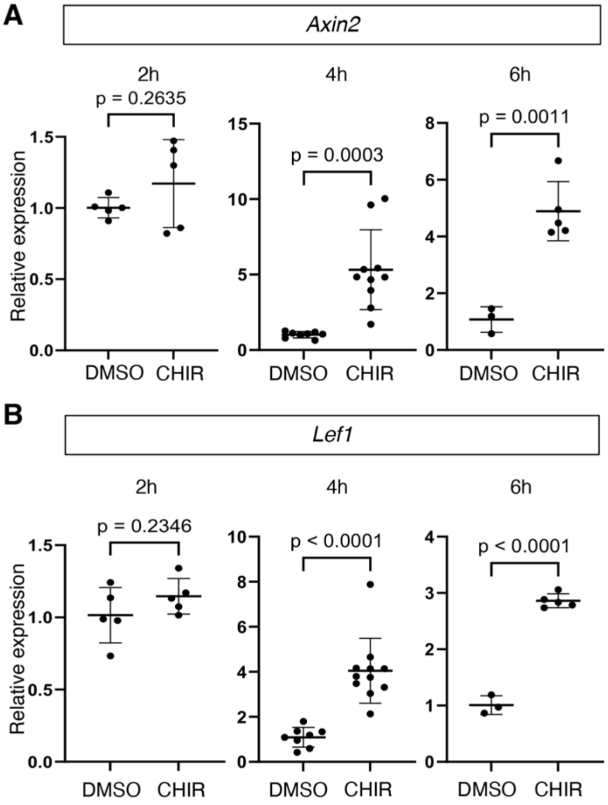

Fig. S6. Expression of *Axin2* and *Lef1* in the E10.5 tail culture experiments

Relative expression levels of *Axin2* (A) and *Lef1* (B) after culturing the tail for indicated times (2, 4, or 6 hours) with CHIR, compared to DMSO.

Each dot represents a tail, and p values by unpaired t-test are shown in each panel.

Development • Supplementary information

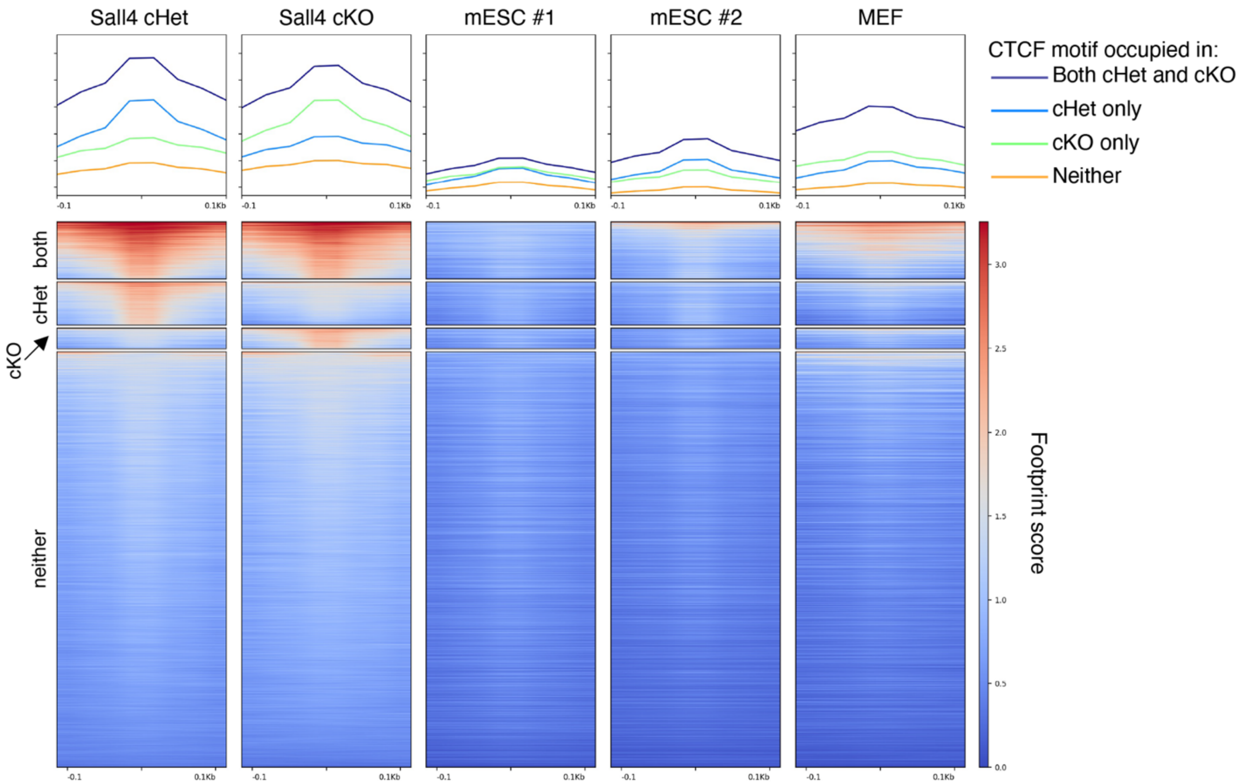

Fig. S7. Heatmap of ATAC-seq data after TOBAIS analysis

Heat map of the CTCF motifs in the *Sall4* cHet posterior trunk, *Sall4* cKO posterior trunk, WT mouse embryonic stem cells in two independent studies (mESC #1 and #2) and WT mouse embryonic fibroblasts (MEF). The x-axis corresponds to ± 100 base pairs from the center of accessible regions (shown as 0). The *Sall4* cHet and *Sall4* cKO heatmaps are the same as Fig. 6C.

Table S1. Genomic regions with enriched SALL4 binding in the posterior trunk mesoderm

Available for download at
https://journals.biologists.com/dev/article-lookup/doi/10.1242/dev.202649#supplementary-data

Table S2. Regions with enriched SALL4 binding in and around select genes related to this study

Available for download at
https://journals.biologists.com/dev/article-lookup/doi/10.1242/dev.202649#supplementary-data

Table S3. Differentially accessible regions between *Sall4* cHet and *Sall4* cKO posterior trunk mesoderm

Available for download at
https://journals.biologists.com/dev/article-lookup/doi/10.1242/dev.202649#supplementary-data

Table S4. Results of gene ontology analysis of genes nearest to differentially accessible regions between *Sall4* cHet and *Sall4* cKO posterior trunk mesoderm

Available for download at
https://journals.biologists.com/dev/article-lookup/doi/10.1242/dev.202649#supplementary-data

Table S5. Results of gene ontology analysis of differentially expressed genes between WT and *Sall4* cKO posterior trunk tissue

Available for download at
https://journals.biologists.com/dev/article-lookup/doi/10.1242/dev.202649#supplementary-data

Table S6. CTCF occupancy scores of CTCF binding motifs in *Sall4* cKO and *Sall4* cHet samples from footprinting analysis of ATAC-seq data

Available for download at
https://journals.biologists.com/dev/article-lookup/doi/10.1242/dev.202649#supplementary-data

Table S7. Results of gene ontology analysis of genes closest to preferentially occupied CTCF sites in *Sall4* cHet relative to *Sall4* cKO posterior trunk mesoderm

Available for download at
https://journals.biologists.com/dev/article-lookup/doi/10.1242/dev.202649#supplementary-data

Development • Supplementary information

Table S8. Primers used for qPCR

Gene	Reference sequence	Primers (5’->3’)
Actb	NM_007393	TGTTACCAACTGGGACGACA<br>CTGGGTCATCTTTTCACGGT
Axin2	NM_015732	TGACTCTCCTTCCAGATCCCA<br>TGCCCACACTAGGCTGACA
Efnb2	NM_010111	ATTATTTGCCCCAAAGTGGACTC<br>GCAGCGGGGTATTCTCCTTC
Epha4	NM_007936	TGGAATTTGCGACGCTGTCA<br>CACTTCCTCCCACCCTCCTT
Gdf11	NM_010272	CCGGCGTCACATCCGTATC<br>ACTTGCTTGAAGTCGATGCTC
Hoxb13	NM_008267	TCTTGCCGAGTATCCAGGAG<br>GGAGTTTCCGAAGGGTAGGTAG
Hoxc13	NM_010464	GCCGTCTACACGGACATCC<br>CCCCAAATGGGTAACCATAGC
Lef1	NM_010703	GCCACCGATGAGATGATCCC<br>TTGATGTCGGCTAAGTCGCC
Lin28a	NM_145833	TGGTGTGTTCTGTATTGGGAGT<br>AGTTGTAGCACCTGTCTCCTTT
Mesp2	NM_008589	CGGCGTTCTCTCACCGATG<br>CACCCCACTACTCATGGCTG
Msgn1	NM_019544	CTTCTGACACCGCTGGTCTG<br>GTGACTGCCGTAGCCATCG
Ripply2	NM_001379243	CCTGGAGAGAAAGAGAAGCG<br>ATAGCCTGACAGGGTGTTGG
Tbx6	NM_011538	ATGTACCATCCACGAGAGTTGT<br>GGTAGCGGTAACCCTCTGTC
Tcf15	NM_009328	GTGTAAGGACCGGAGGACAA<br>GATGGCTAGATGGGTCCTTG

Development • Supplementary information
