# Supplementary material for: Sall4 regulates posterior trunk mesoderm development by promoting mesodermal gene expression and repressing neural genes in the mesoderm
Source: Development. 2024 Mar 4;151(5):dev202649. doi: 10.1242/dev.202649 (PMC10946440; doi:10.1242/dev.202649)
Supplement: Supplementary file 1 [file develop-151-202649_review_history.pdf]

Sall4 regulates posterior trunk mesoderm development by promoting mesodermal gene expression and repressing neural genes in the mesoderm

Matthew P. Pappas, Hiroko Kawakami, Dylan Corcoran, Katherine Q. Chen, Earl Parker Scott, Julia Wong, Micah D. Gearhart, Ryuichi Nishinakamura, Yasushi Nakagawa and Yasuhiko Kawakami
DOI: 10.1242/dev.202649

Editor: Haruhiko Koseki

Review timeline

Original submission:	21 November 2022
Editorial decision:	20 December 2022
Resubmission:	28 December 2023
Accepted:	30 January 2024

Original submission

First decision letter

MS ID#: DEVELOP/2022/201465

MS TITLE: Sall4 regulates posterior trunk mesoderm development by promoting mesodermal gene expression and chromatin accessibility that promotes WNT signaling and represses neural genes within the mesoderm

AUTHORS: Matthew P. Pappas, Hiroko Kawakami, Katherine Q. Chen, Dylan Corcoran, Earl Parker Scott, Julia Wong, Micah D. Gearhart, Ryuichi Nishinakamura, Yasushi Nakagawa, and Yasuhiko Kawakami

ARTICLE TYPE: Research Article

Dear Dr. Kawakami,

I have now received all the referees reports on the above manuscript, and have reached a decision. The referees' comments are appended below, or you can access them online: please go to BenchPress and click on the 'Manuscripts with Decisions' queue in the Author Area.

As you will see from their reports, the referees recognise the potential of your work, but they also raise significant concerns about it. Given the nature of these concerns, I am afraid I have little choice other than to reject the paper at this stage.

However, having evaluated the paper, I do recognise the potential importance of this work. Particularly, it would be great if the model could be validated by whatever experimental perturbation. I would therefore be prepared to consider as a new submission an extension of this study that contains new experiments, data and discussions and that address fully the major concerns of the referees. The work required goes beyond a standard revision of the paper. Please bear in mind that the referees (who may be different from the present reviewers) will assess the novelty of your work in the context of all previous publications, including those published between now and the time of resubmission.

© 2024. Published by The Company of Biologists under the terms of the Creative Commons Attribution License (https://creativecommons.org/licenses/by/4.0/).

Reviewer 1

Advance summary and potential significance to field

This manuscript describes an important advance in our understanding of how the zinc-finger transcription factor Sall4 regulates posterior mesodermal development by activating genes needed for mesodermal (somite) development and repressing genes for neural development. They found that a Sall4 conditional KO results in loss of expression of many genes needed for mesoderm development and somite formation such as Msgn1, Mesp2, Ripply2, Eph4, and Uncx as well as activators of Wnt, FGF, and retinoic acid signaling. By combining chromatin ATAC-seq studies with RNA-seq studies on WT vs Sall4 mutants, they found that loss of Sall4 reduces chromatin accessibility near many of the genes that showed reduced expression, suggesting that Sall4 regulates some genes directly. In addition, they report that Sall4 represses some genes potentially directly by recruiting the repressive factor CTCF. Overall, these studies provide important new findings that help us better understand how undifferentiated cells emerging from the posterior growth region decide to differentiate into mesoderm rather then neuroectoderm.

Comments for the author
Specific Points:
1. Abstract line 19 - In the last sentence it might be good to say that Sall4 regulates paraxial mesoderm differentiation by directing activation of mesodermal genes and repression of neural genes.
2. Line 238 - Do the authors have any in situ hybridization data to show that neural genes are misexpressed in trunk mesoderm in the Sall4 KO? Or that neural genes are normally expressed in spinal cord?
3. Line 261 - In addition to identification of CTCF as a repressive factor recruited by Sall4, was there any attempt to determine if Sall4 regulates recruitment of the repressive factor Polycomb or the repressive histone mark H3K27me3?
4. Discussion - Since Sall4 is expressed in both trunk mesoderm and spinal cord, the authors need to address how Sall4 could repress neural gene expression in mesoderm, but not in spinal cord.
5. Minor point - In Fig. 2 graphic the word Delta is misspelled as Delat.

Reviewer 2

Advance summary and potential significance to field

This manuscript builds on previous work from the lab on a cKO of Sall4, which leads to a phenotype posterior to the lumbar level with truncation and a disorganised axial skeleton. The expression patterns of genes known to be important in paraxial mesoderm are examined in WT and cKO embryos at E9.5 and E10.5. To gain insights into Sall4 function the authors performed ATAC-seq of posterior mesoderm, comparing cKO to heterozygous +/fl animals. They find that chromatin accessibility is reduced in absence of Sall4. There is a correlation with regulators of Wnt signalling and one gene, Cxxc4, is assessed directly by RNAscope. It is puzzling that accessibility is reduced but expression increased for the selected genes shown. Of 1701 differentially accessible regions, they find 17 sequences located close to genes whose functions are associated with neural development. The functional significance is not clear, or whether this is a meaningful correlation. The majority of the findings presented are associations, which could be interesting, but do not provide a mechanistic explanation. This is a limitation of the study.

Comments for the author

additional information that should be provided:
Please state what somite level is being assessed at E9.5 and E10.5 for gene expression changes. The cKO has a phenotype posterior to the lumbar level.
Fig1. DISH for at least one of the PM/PSM marker with Uncx4 in mutant embryos would better assess the expression pattern. Expression of Sall4, or lack thereof, should also be shown in the mutant.

© 2024. Published by The Company of Biologists under the terms of the Creative Commons Attribution License (https://creativecommons.org/licenses/by/4.0/).

Fig3. Uncx4 expression narrowed along the medio-lateral axis in Sall4 (line 154). This has not been quantified and it is difficult to see at E9.5, the disorganised and weaker pattern becomes clear at E10.5. Other 'narrowing' is also difficult to assess, e.g. Pax3, Pax9 - it may just be weaker.
Fig4. ATAC-seq should also be performed in WT embryos. Explain the mesoderm-enriched fraction that was used, it is not enough to say "similar to our previous experiment" (line 185).
Fig. 5 RNAscope could use multiplexing to assess additional gene expression.
minor:
line 50, skeletal muscles of the trunk and limbs
line 169/170, SALL4 (protein?!) versus Sall4 (not capitalised) directly regulates

<u>Reviewer 3</u>

Advance summary and potential significance to field

In this manuscript Pappas et al. describe a role for Sall4 in regulating posterior trunk mesoderm development by promoting the expression of mesodermal genes and suppressing the expression of Wnt repressors and pro-neural factors in the mesoderm. Using in situ data the authors show that the expression of genes involved in somitogenesis are disrupted in the absence of Sall4. Using genomic data the authors further argue that Sall4 promotes Wnt signaling by regulating chromatin accessibility of Wnt activators and repressors, and promotes mesodermal differentiation by indirectly repressing pro-neural genes via CTCF. This manuscript provides further characterization of the Sall4 conditional knockout previously described by the same group; however, the data shown are largely correlative, and there are several major issues that the authors fail to address. The paper has a “patchwork” feel, with the first half being largely descriptive in situ gene expression analyses, and the second half (the genomic data), ostensibly addressing mechanism.
However, the gene expression analyses did not provide novel insights into somitogenesis or Sall4 function, and the genomic data that is presented seems to have little to no connection to the in situ data. None of the changes in the expression of Wnt signaling components, suggested by the genomic analyses, were validated by in situ analyses. More importantly, functional studies supporting a role for CTCF or Wnt signaling downstream of Sall4 were not presented. As it stands, this work does not provide significant insights or advances to the field.

Comments for the author

Major comments

1. Throughout the manuscript the authors refer to stages E9.5 and E10.5. They state that Sall4 is knocked out in the TCre line beginning at stage E7.5, but that the severe phenotype does not appear until stage E10.5 due to persistence of the Sall4 protein. Previous work by the same group shows that at E8.5 Sall4 expression is lost in the trunk mesoderm (Supplementary data, Tahara et al. 2019). However, it appears that expression persists in the lateral epiblast cells at this stage. As neuromesodermal progenitor cells contribute to trunk mesoderm, it is difficult to be sure that Sall4 is knocked out in the mesoderm at E9.5 and E10.5 without performing lineage tracing experiments for TCre recombination in the TCre;Sall4 cKO line, for example using a Rosa-LacZ or Rosa-MTMG background. This is necessary to confirm that the tissue observed is actually negative for Sall4 expression. Further, as previous work shows that expression of Sall4 in the mesoderm is absent at E8.5, it is likely that the expression of genes investigated in this manuscript are also affected at this earlier stage. The authors should consider looking at the expression patterns of their genes of interest at E8.5. As the authors note, by E9.5 the mutants are already distinguishable by a shorter and thinner posterior trunk, suggesting that phenotypic changes have already manifested.

2. The authors suggest that Sall4 maintains the expression of segmentation clock genes and show that the expression of cycling genes Delta1 and Notch1 is reduced at E9.5 and severely disrupted at E10.5. However, as the expression at only one timepoint is shown, these data do not indicate that the cycling expression of clock genes is affected, and as such the authors cannot state that the segmentation clock is affected. This can be resolved by increasing the number of embryos in the experiment or by performing embryo half culture experiments to observe the expression of these genes at various points in their expression cycle.

© 2024. Published by The Company of Biologists under the terms of the Creative Commons Attribution License (https://creativecommons.org/licenses/by/4.0/).

3. The observed phenotype becomes much more severe between E9.5 and E10.5, which, as the authors note, coincides with the trunk to tail transition. As such, the authors should explore whether the trunk to tail transition is disrupted in TCre;Sall4 cKO embryos by investigating expression of Gdf11 and other trunk to tail markers.

4. There are several instances where the in situ data does not support the authors' claims. The expression domains shown by several in situs are clearly smaller in the cKO embryos relative to the WT embryos (e.g. Mesp2 and Ripply2); however, it is unclear whether this is a secondary effect from an overall smaller tissue volume in the tail in the cKO embryos. As such, it is inappropriate to assume that the smaller domain is due to regulation of the gene of interest by Sall4. The authors also state that Mesp2 displays reduced signal intensity in cKO embryos at E9.5 relative to the WT, and that Uncx4 expression is narrower in cKO embryos; however, neither of these claims is clearly shown by the in situ data.

5. The authors argue that Sall4 promotes Wnt signaling by regulating accessibility of chromatin at Wnt activators and repressors. The results used to support this claim are largely correlative, and do not clearly demonstrate that Wnt signaling is affected by Sall4 activity. To strengthen their argument, the authors should consider performing in situs to observe expression of targets of Wnt signaling in the embryo, as well as functional manipulation experiments to determine whether (over)activation of Wnt signaling in the cKO embryo is sufficient to rescue the phenotype.

6. To determine whether differential CTCF peaks are specific to posterior trunk mesoderm the authors compare CTCF occupancy, as inferred from ATAC-seq data, to previously published CTCF ChIP-seq data obtained from mESCs and MEFs. This methodology presents several problems:
a. The authors state that differential CTCF peaks are specific to posterior trunk mesoderm using CTCF peaks mapped to undifferentiated mESCs and MEFs. However, to fully determine whether peaks are specific to mesoderm, the authors should compare their peaks to data generated from mESCs differentiated to neural and mesodermal fates, in addition to undifferentiated mESCs. If the peaks are then solely correlated with the mesodermal cells the authors can more convincingly determine the specificity of CTCF binding peaks to posterior trunk mesoderm.
b. The authors compare CTCF occupancy data inferred from ATAC-seq to CTCF ChIP-seq data. This is not an adequate comparison to determine the specificity of differential CTCF peaks to posterior trunk mesoderm tissue. For the data modalities to be truly comparable, the authors should process previously published mESC and MEF ATAC-seq data using the same TOBIAS method used to calculate inferred CTCF occupancy and compare this inferred occupancy with their data. Conversely, the authors could perform CTCF ChIP-seq analysis of their cHet and cKO samples.

Minor comments
1. On page 11, line 279, it is unclear what the term "target genes" is referring to.
2. In figure 2, Delta1 is misspelled as Delat1.

Response to the reviewers

Thank you for the critical and helpful comments regarding our manuscript (DEVELOP/2022/201465) entitled "Sall4 regulates posterior trunk mesoderm development by promoting mesodermal gene expression and chromatin accessibility that promotes WNT signaling and represses neural genes within the mesoderm." We have examined the comments carefully and revised the manuscript and figures with new images from additional experiments and additional bioinformatics analyses. New data are presented in 57 new panels, which are embedded in 3 main figures and 6 supplemental figures. Major changes in the figures include:
(1) rescue of gene expression in *Sall4* cKO tail by activating ß-catenin signaling (Fig. 5H-L'); (2) qRT-PCR analysis of *Mesp2*, *Ripply2*, *Epha4* and *Efnb2* in the posterior trunk at E9.5 (Fig. 1B"- E"); (3) An additional RNAscope for *Axin2* and *Rspo2* (Fig. 5C-G); and (4) new TOBIAS footprinting analysis (Fig. 6C, S7). All of the new data are consistent with our initial findings. We believe that the additional experiments and computational analyses adequately address the Reviewers' concerns and significantly strengthened the revised manuscript. Each of the reviewers' comments and point-by-point answers are provided below. Our responses are described with blue font.

© 2024. Published by The Company of Biologists under the terms of the Creative Commons Attribution License (https://creativecommons.org/licenses/by/4.0/).

Please note that the comments are numbered continuously.

Response to Reviewer 1
We thank reviewer 1 for stating that "this manuscript describes an important advance in our understanding of how the zinc-finger transcription factor Sall4 regulates posterior mesodermal development". We are happy to know that reviewer 1 believed that these studies "provide important new findings that help us better understand how undifferentiated cells emerging from the posterior growth region decide to differentiate into mesoderm rather than neuroectoderm". Below are our responses.

Comment 1. Abstract line 19 - In the last sentence it might be good to say that Sall4 regulates paraxial mesoderm differentiation by directing activation of mesodermal genes and repression of neural genes.

Response: We thank the reviewer for this suggestion.
We have changed the last sentence of the abstract as below (line 19-21).

> Our study unveiled multiple mechanisms by which *Sall4* regulates paraxial mesoderm development by directing activation of mesodermal genes and repression of neural genes.

Comment 2. Line 238 - Do the authors have any in situ hybridization data to show that neural genes are misexpressed in trunk mesoderm in the Sall4 KO? Or that neural genes are normally expressed in spinal cord?

Response: In our in situ hybridization data, we did not detect misexpression of neural genes (*Sox2*, *Sox1*) in the trunk mesoderm. We also examined SOX2 immunofluorescence on sections at E9.5. As shown below, we detected a few SOX2-positive cells outside of the neural ube (n=3). We also examined expression of FOXA2 (n=6), NKX2.2 (n=6), OLIG2 (n=6), and ISL1 (n=6) by immunostaining; however, we did not detect ectopic expression of these markers.

Although *Sall4* cKO caused elevated neural programs in the mesoderm by RNA-seq in our previous study (Tahara et al. Development, 2019. PMID: 31235634), we speculate that elevated neural program in the posterior trunk mesoderm is not strong enough to broadly induce ectopic expression of neural markers.

We chose not to include this data in the manuscript. Investigation of ectopic neural gene expression in the mesoderm would require more thorough examination. We think such a study is beyond the focus of this manuscript.

NOTE: We have removed unpublished data that had been provided for the referees in confidence.

SOX2 immunofluorescent staining on WT and *Sall4* cKO section (E9.5). Small number of ectopic SOX2 signals (arrowheads) were observed in the *Sall4* cKO embryo. g: gut tube, nt: neural tube.

Regarding neural gene expression in the spinal cord, we observed accelerated neural gene expression in *Sall4* cKO embryos in our previous study (Tahara et al. Development, 2019. PMID: 31235634). We observed that NKX2.2 and ISL1 are expressed in *Sall4* cKO earlier than WT embryos at the same axial level at the same stage. We briefly described this information in the discussion section (line 436-438).

Comment 3. Line 261 - In addition to identification of CTCF as a repressive factor recruited by Sall4, was there any attempt to determine if Sall4 regulates recruitment of the repressive factor Polycomb or the repressive histone mark H3K27me3?

Response: We agree that investigating whether and how SALL4 recruits other repressive factors, such as polycomb factors and repressive histone marks, is important. Such a study would require a new series of ChIP-seq and/or CUT&RUN/CUT&Tag experiments, and we think that such experiments are important next step from the current study. We appreciate this comment as it provides us with an important future direction of our study.

Comment 4. Discussion - Since Sall4 is expressed in both trunk mesoderm and spinal cord, the

© 2024. Published by The Company of Biologists under the terms of the Creative Commons Attribution License (https://creativecommons.org/licenses/by/4.0/).

authors need to address how Sall4 could repress neural gene expression in mesoderm, but not in spinal cord.

Response: Our data suggest that SALL4 represses the neural program in the mesoderm tissue. In the neural tissue, we think that SALL4 also represses neural program, but it does notcompletely suppress the high levels of the neural gene network as compared to the mesoderm tissue. This idea is based on the accelerated expression of ISL1 in the neural tube of *Sall4* cKO embryos, as mentioned in response to comment 3.

There are several possible reasons for the different degrees of restriction or repression of neural gene expression in different contexts. We think that tissue-specific SALL4 behavior is a compelling explanation. For example, while SALL4 directly binds to AT-rich sequences in mouse embryonic stem cells (Pantier et al., 2021; Ru et al., 2022), SALL4 indirectly binds to DNA via its interaction partner PLZF in spermatogonial stem cells (Lovelace et al., 2016). The presence or absence of SALL4 molecular partners may explain the different degrees of repression of neural gene expression in mesodermal vs neural cells.

We have included this short discussion in the discussion section (line 446- 459).

Comment 5. Minor point - In Fig. 2 graphic the word Delta is misspelled as Delat.
Response: We have corrected this mistake.

Response to Reviewer 2
Thank you for the comments, which helped us improve our manuscript. Below, we have our responses to each comment.

Comment 6: Please state what somite level is being assessed at E9.5 and E10.5 for gene expression changes. The cKO has a phenotype posterior to the lumbar level.

Response: We used 24 – 28 somite embryos for E9.5 and 34 - 39 somite embryos for E10.5. The in situ hybridization images show the posterior part of these embryos.
We have included this information in line 105-106.

Comment 7: Fig1. DISH for at least one of the PM/PSM marker with Uncx4 in mutant embryos would better assess the expression pattern. Expression of Sall4, or lack thereof, should also be shown in the mutant.

Response: The expression patterns of the PM/PSM genes used in this study have been well characterized. Given that *Uncx4* expression is weaker in *Sall4* cKO embryos than in WT embryos, double in situ of PM/PSM gene with *Unxc4* would not provide new information beyond the lower levels of expression in *Sall4* cKO embryos, which is already shown.

We wondered if Reviewer 2 made this comment because the *Sall4* expression pattern was not clear to Reviewer 2. Therefore, we show below another *Sall4* in situ image (labelled as A'"), in which the color reaction of the in situ was not stopped early (compared to double color in situ, in which *Sall4* color was stopped early: labelled as A-A"). The image clearly shows the *Sall4* expression domain along with the somite boundary.

In response to this critique, we included a panel with strong *Sall4* signals in Figure 1 to help readers understand the *Sall4* expression pattern (Fig. 1A'"). We have also included a panel showing loss of *Sall4* transcripts in *Sall4* cKO tail (Fig. 1A"").

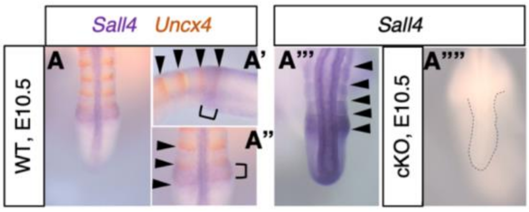

© 2024. Published by The Company of Biologists under the terms of the Creative Commons Attribution License (https://creativecommons.org/licenses/by/4.0/).

Comment 8: Fig3. Uncx4 expression narrowed along the medio-lateral axis in Sall4 (line 154). This has not been quantified and it is difficult to see at E9.5, the disorganised and weaker pattern becomes clear at E10.5. Other 'narrowing' is also difficult to assess, e.g. Pax3, Pax9 - it may just be weaker.

Response: We agree that "narrowing" of expression domain is not visibly evident at E9.5. Reviewer 3 also raised a similar concern (Comment 16). To clarify whether the expression domain is truly narrower, we quantified the width of the most posterior expression domain along the medio-lateral axis by FIJI image analysis.
As shown below, the width of expression domain of *Uncx4*, *Pax3*, and *Pax9* was narrower in *Sall4* cKO, compared to WT.

We are thankful for the reviewer's comment, which helped us clarify the somite phenotype at E9.5 with this analysis. We have included these results as new Fig. S4, and described the results in line 168 – 170 and 191-192.

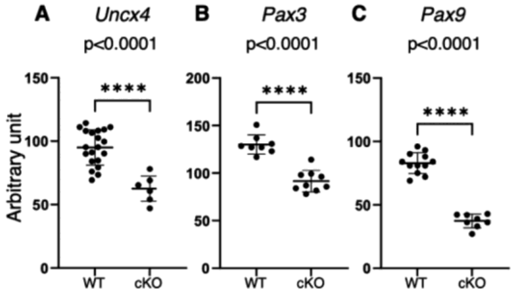

Comment 9: Fig4. ATAC-seq should also be performed in WT embryos. Explain the mesoderm-enriched fraction that was used, it is not enough to say "similar to our previous experiment" (line 185).

Response: In the ATAC-seq experiment, we used *TCre*$^{tg/+}$; *Sall4*$^{flox/+}$ conditional heterozygous (cHet) embryos as a control. If we compare WT and cHet samples, there would likely be differences in gene expression levels and chromatin accessibility. However, such differences alone are unlikely to drive the *Sall4* cKO phenotype, given that *Sall4* cHet embryos are essentially normal with respect to morphology.

The purpose of the ATAC-seq experiment is to determine differentially accessible regions involved in the phenotype of *Sall4* cKO embryos. Moreover, we can obtain both cHet and cKO from the same litter, since our timed mating scheme is *Sall4*$^{flox/flox}$ females x *TCre*$^{tg/tg}$; *Sall4*$^{flox/+}$ males. We reasoned that using littermates as controls would lower experimental noise. Additionally, we believed that the differences detected between cHet and cKO would be related to the *Sall4* cKO phenotype.

Indeed, our analysis successfully identified differentially accessible regions between *Sall4* cKO and *Sall4* cHet control, which led to identification of WNT/ß-catenin signaling and CTCF occupancy as *Sall4* downstream mechanisms. Therefore, we believe that using *Sall4* cHet as a control sample is appropriate in our ATAC-seq experiments.

Regarding the mesoderm-enriched fraction that we prepared for ATAC-seq experiment, the following description was included under "ATAC-seq" in the Material and Methods section in the original manuscript.

> "Tissues posterior to the boundary of PSM and the somite were collected and kept in PBS on ice during dissection. The neural tube was removed as previously described (Tahara et al., 2019). The remaining tissue (the posterior mesoderm tissue) was dissociated by TrypLE (Invitrogen) at 37°C for 5 minutes, neutralized by DMEM + 10% FBS, and collected by low- speed centrifugation."

In order to address the concern, we have provided more detailed procedures as below. The underlined part is a newly added information. We have updated the Materials and Methods section with the new information.

© 2024. Published by The Company of Biologists under the terms of the Creative Commons Attribution License (https://creativecommons.org/licenses/by/4.0/).

Line 516 – 521: "Tissues posterior to the boundary of PSM and the somite were collected and kept in PBS on ice during dissection. The dissected tissues were treated by dispase (1.5mg/ml. Roche, 4942078001, 37°C, 5min), followed by removal of the neural tube using a tungsten needle in cold PBS, as previously described (Tahara et al., 2019). The remaining tissue (the posterior mesoderm tissue) was dissociated by TrypLE (Invitrogen) at 37°C for 5 minutes, neutralized by DMEM + 10% FBS, and collected by low-speed centrifugation (500xg for 3 minutes at room temperature)."

Comment 10: Fig. 5 RNAscope could use multiplexing to assess additional gene expression.

Response: Thank you for this suggestion. Although the reviewer did not suggest specific genes to be examined, we have included images of *Axin2*, which is a target of WNT signaling. As expected, expression of *Axin2* showed a clear reduction in the PSM of *Sall4* cKO embryos.

In addition, we have also examined the expression pattern of *Rspo2*. Consistent with our RNA-seq analysis data in Fig. 5A, *Rspo2* showed reduced expression in the paraxial mesoderm of *Sall4* cKO embryos.

We have included these new data in Fig. 5C, C', E, E', and F, as shown below. We have described this new data in line 253 – 254 and 269 – 271.

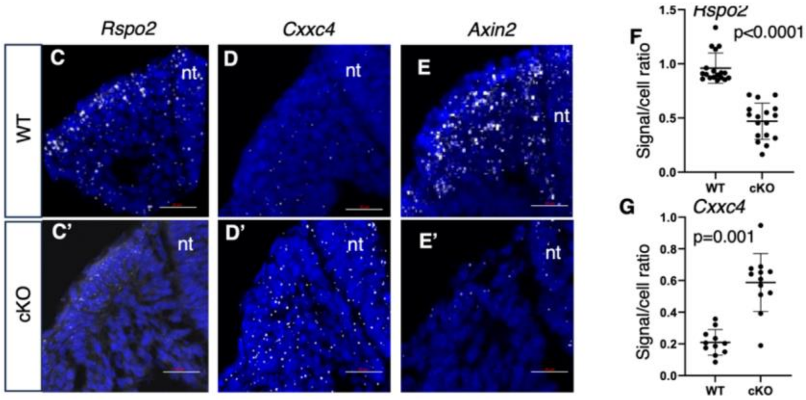

minor:
Comment 11: line 50, skeletal muscles of the trunk and limbs
Response: We have corrected this (line 51).

Comment 12: line 169/170, SALL4 (protein?!) versus Sall4 (not capitalised) directly regulates
Response: We have changed *Sall4* to SALL4 (line 186).

Response to Reviewer 3
Thank you for the comments, which helped us to improve our manuscript. Below, we have our responses to each comment.

Major comments
Comment 13-1: 1. Throughout the manuscript the authors refer to stages E9.5 and E10.5. They state that Sall4 is knocked out in the TCre line beginning at stage E7.5, but that the severe phenotype does not appear until stage E10.5 due to persistence of the Sall4 protein. Previous work by the same group shows that at E8.5 Sall4 expression is lost in the trunk mesoderm (Supplementary data, Tahara et al. 2019). However, it appears that expression persists in the lateral epiblast cells at this stage. As neuromesodermal progenitor cells contribute to trunk mesoderm, it is difficult to be sure that Sall4 is knocked out in the mesoderm at E9.5 and E10.5 without performing lineage tracing experiments for TCre recombination in the TCre;Sall4 cKO line, for example using a Rosa-LacZ or Rosa-MTMG background. This is necessary to confirm that the tissue observed is actually negative for Sall4 expression.

Response: We want to clarify that we are not investigating neuromesodermal progenitor cells in the lateral epiblast or chordo-neural hinge in this study. As the reviewer wrote, we have shown that SALL4 protein was still detected in the lateral epiblast, where NMPs are located, at E9.5 in

© 2024. Published by The Company of Biologists under the terms of the Creative Commons Attribution License (https://creativecommons.org/licenses/by/4.0/).

TCre; Sall4 cKO embryos (Tahara et al., Development 2019 PMID: 31235634).

In this manuscript, we investigate roles of *Sall4* in the paraxial mesoderm, where SALL4 protein is largely undetectable (please see below). Moreover, we show below *TCre; R26-LacZ* images, which show robust and broad recombination by *TCre*, which demonstrates that the posterior trunk mesoderm is derived from the *TCre* lineage, in which *Sall4* is knocked out.

We routinely include SALL4 in immunofluorescence experiments. Below, we show immunofluorescence images acquired at the same conditions after slides were stained with the same conditions. With the setting to detect residual SALL4 signals in *Sall4* cKO embryos, the SALL4 signals in the WT embryos were essentially saturated. The images clearly demonstrate loss of SALL4 immunoreactivities from the paraxial mesoderm.

Rather than the suggested lineage analysis, these data clearly show that the *Sall4* gene is recombined by *TCre* in the tissue examined in this study and that only residual SALL4 is detectable in the paraxial mesoderm of the posterior trunk of *Sall4* cKO embryos.

NOTE: Figure provided for reviewer has been removed. It showed Figure S2A from Akiyama, R., Kawakami, H., Wong, J., Oishi, I., Nishinakamura, R. and Kawakami, Y. (2015). *Sall4-Gli3* system in early limb progenitors is essential for the development of limb skeletal elements. *Proc Natl Acad Sci USA*. 112 (16), 5075-5080. doi: 10.1073/pnas.1421949112

Please hyperlink the doi to "https://www.pnas.org/doi/full/10.1073/pnas.1421949112"

LacZ stanning of *TCre; R26-LacZ* embryos (from Akiyama et al., PNAS 2015, PMCID: PMC4413345). Recombination by *TCre* can be detected as early as E7.5, and the cells recombined by *TCre* broadly contribute to the post-cranial tissues.

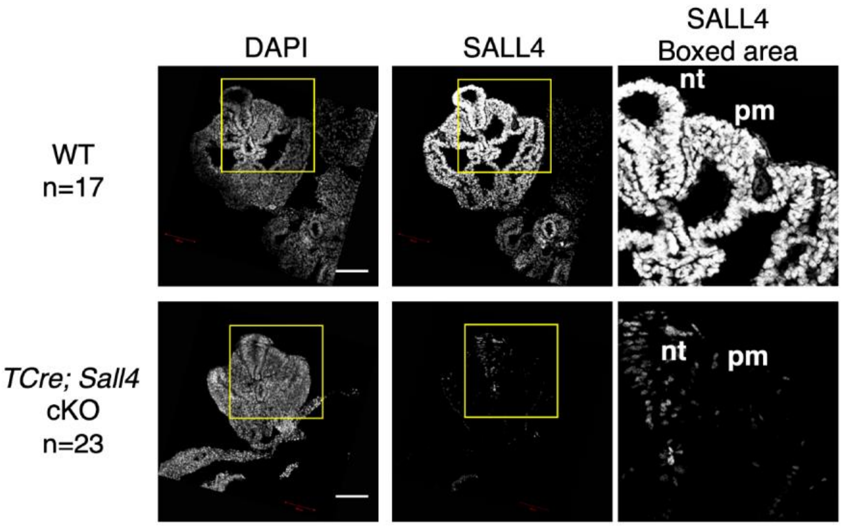

SALL4 immunofluorescence on cross sections of the posterior trunk of WT and *Sall4* cKO embryos at E9.5-9.75. SALL4 immunoreactivities are significantly lost in the posterior trunk tissues, including the paraxial mesoderm.

We have modified the text in line 102 – 103 to describe robust recombination by *TCre* and cited our previous paper (Akiyama et al., PNAS 2015, PMCID: PMC4413345) that shows the *TCre; R26-LacZ* data above. We have also included SALL4 immunofluorescence in *Sall4* cKO embryos as Figure S1.

Comment 13-2: Further, as previous work shows that expression of Sall4 in the mesoderm is absent at E8.5, it is likely that the expression of genes investigated in this manuscript are also affected at this earlier stage. The authors should consider looking at the expression patterns of their genes of interest at E8.5. As the authors note, by E9.5 the mutants are already distinguishable by a shorter and thinner posterior trunk, suggesting that phenotypic changes have already manifested.

Response: As suggested by the reviewer, we examined expression of *Mesp2*, *Ripply2*, *Epha4* and *Efnb2* by qRT-PCR using the trunk tissue of E8.5 embryos. We chose qRT-PCR because it is more

© 2024. Published by The Company of Biologists under the terms of the Creative Commons Attribution License (https://creativecommons.org/licenses/by/4.0/).

sensitive than in situ hybridization. These genes exhibited reduced expression levels compared to WT embryos, which we now show in Fig. S3. The degree of downregulation at E8.5 seems to be milder than that of E9.5 (shown in Fig. 1B”-E”). E8.5 embryos (8 - 10 somite stage) already developed the trunk to the future thoracic levels, and we did not observe thoracic vertebrae defects in *Sall4* cKO mutants at P0 (Tahara et al., Development 2019 PMID: 31235634). In contrast, E9.5 embryos (24-28 somite stage) developed the trunk towards the future lumbar/sacral levels, and we observed vertebrae defects at the lumbar/sacral levels. These observations indicate that expression levels of genes involved in PSM differentiation are already impaired at E8.5 in *Sall4* cKO embryos and that the degree of reduction of gene expression correlates with posterior axial skeletal defects.

We have included above mentioned qRT-PCR data in Fig. 1B”-E” (E9.5 data) and Fig. S3 (E8.5 data). We have also included this description in line 155-163.

Comment 14: The authors suggest that Sall4 maintains the expression of segmentation clock genes and show that the expression of cycling genes Delta1 and Notch1 is reduced at E9.5 and severely disrupted at E10.5. However, as the expression at only one timepoint is shown, these data do not indicate that the cycling expression of clock genes is affected, and as such the authors cannot state that the segmentation clock is affected. This can be resolved by increasing the number of embryos in the experiment or by performing embryo half culture experiments to observe the expression of these genes at various points in their expression cycle.

Response: We agree that we cannot state that the segmentation clock is affected without more detailed analysis.
What we wanted to describe is the observed changes of gene expression pattern in *Sall4* cKO embryos. We realized that our description was misleading, and it appeared that we wanted to stress the function of *Sall4* in segmentation clock regulation, which is not our focus.
To clarify this point and to avoid misleading descriptions, we have changed our description in line 354-356 as below, and revised Figure 7.
In the PSM, *Sall4* contributes to regulation of WNT and FGF signaling and helps maintain the expression of genes involved in Notch signaling and somite boundary formation (Fig. 7A).

Comment 15: observed phenotype becomes much more severe between E9.5 and E10.5, which, as the authors note, coincides with the trunk to tail transition. As such, the authors should explore whether the trunk to tail transition is disrupted in TCre;Sall4 cKO embryos by investigating expression of Gdf11 and other trunk to tail markers.

Response: We thank the reviewer for this comment, which helped us to investigate the possibility whether the trunk to tail transition is affected in *Sall4* cKO mutants.

As suggested, we have performed *Gdf11* in situ. We have also performed *Lin28a* in situ. These markers are used by the group of Dr. Moises Mallo (Jurberg et al., 2013, Dev Cell, PMID: 23763947 and Aires et al., 2018, Dev Cell. PMID: 30661984). We have also examined expression patterns of *Hoxb13* and *Hoxc13* to figure out whether tail bud progenitors are affected.

As shown below, at E9.5, both *Gdf11* and *Lin28a* are expressed in the posterior part of the body, and qRT-PCR analysis indicated that the expression levels of these genes were lower in *Sall4* cKO than in WT. These data suggest that the trunk to tail transition is impaired in *Sall4* cKO embryos, which correlates with vertebrae/tail defects shown in the Tahara et al paper (Development, 2019, PMCID: PMC6679359).
At E10.5, expression of *Gdf11*, *Lin28a*, *Hoxb13* and *Hoxc13* was downregulated as measured by both in situ and qRT-PCR. Given that tail bud progenitor activity relies on a network by *Gdf11*, *Lin28*, *Hoxb13* and *Hoxc13*, these results suggest that severe tail truncation in *Sall4* cKO mutants is, at least in part, derived from reduced activities of tail bud progenitor cells.

© 2024. Published by The Company of Biologists under the terms of the Creative Commons Attribution License (https://creativecommons.org/licenses/by/4.0/).

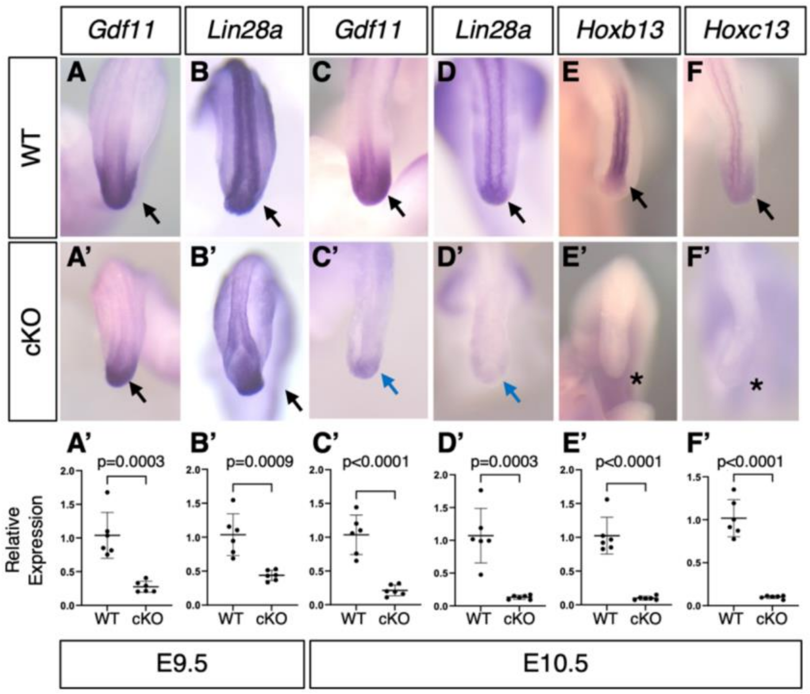

We have described these new data in line 196 - 211. We have also included the new data as Figure S5.

Comment 16: There are several instances where the in situ data does not support the authors' claims. The expression domains shown by several in situs are clearly smaller in the cKO embryos relative to the WT embryos (e.g. Mesp2 and Ripply2); however, it is unclear whether this is a secondary effect from an overall smaller tissue volume in the tail in the cKO embryos. As such, it is inappropriate to assume that the smaller domain is due to regulation of the gene of interest by Sall4. The authors also state that Mesp2 displays reduced signal intensity in cKO embryos at E9.5 relative to the WT, and that Uncx4 expression is narrower in cKO embryos; however, neither of these claims is clearly shown by the in situ data.

Response: Thank you for the comment. We agree that we have to carefully describe the reduced gene expression in relation to small tissue size. The reviewer wrote "*it is inappropriate to assume that the smaller domain is due to regulation of the gene of interest by Sall4*". We would like to clarify that we did not describe that smaller expression domain is derived from reduced gene expression. To avoid misunderstanding, we have changed our description about tissue size and gene expression. Specifically, we have written as below in line 172 – 173.

Original sentence: These expression patterns indicate severe defects in somite development in *Sall4* cKO embryos.

New sentence: These expression patterns indicate severe reduction of the posterior trunk and tail size in *Sall4* cKO embryos.

Regarding the signal intensity of the *Mesp2* and *Uncx4* expression domains, Reviewer 2 also made a similar comment (Comment 8). To address this concern and the issue raised above, we have done two experiments/measurements.

1. We have performed qRT-PCR using RNA prepared from the tissue posterior to the last somite from 24 – 25 somite stage embryos. Compared to WT embryos, expression levels of *Mesp2*, as well as *Ripply2*, *Epha4*, *Efnb2*, are reduced in *Sall4* cKO embryos, at E9.5. We have described this data in line 114 – 116. We have included qRT-PCR results of *Mesp2*, *Ripply2*, *Epha4*, *Efnb2* in Fig. 1B", C", D", and E", as shown below.

© 2024. Published by The Company of Biologists under the terms of the Creative Commons Attribution License (https://creativecommons.org/licenses/by/4.0/).

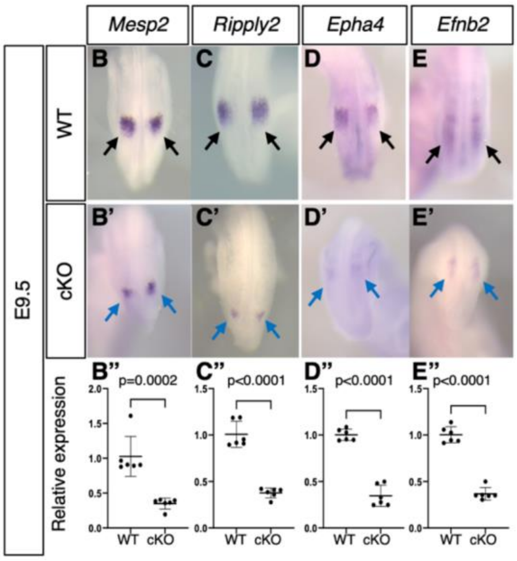

2. We have measured the width of the last (newest) somite using FIJI. The measurement shows narrower expression domains for *Uncx4*, *Pax3*, and *Pax9* in *Sall4* cKO embryos at E9.5.
   We have described this data in line 168 – 170 and line 191 - 192. We have included these new data in new Fig. S4, as below.

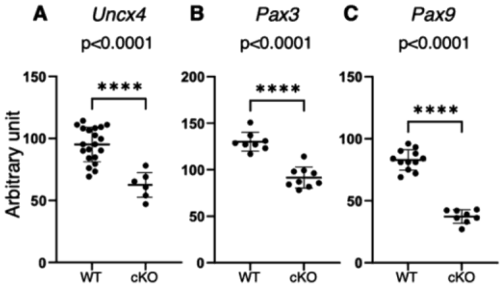

The reviewer commented that “it is unclear whether this (smaller expression domain) is a secondary effect from an overall smaller tissue volume in the tail in the cKO embryos.” We think reduced gene expression levels, as quantified by qPCR, which are normalized to ubiquitously expressed *Actb* (beta-actin) mRNA, correlates with a proportionally smaller expression domain. As described above, we have also changed a description about somite width.

Comment 17: The authors argue that Sall4 promotes Wnt signaling by regulating accessibility of chromatin at Wnt activators and repressors. The results used to support this claim are largely correlative, and do not clearly demonstrate that Wnt signaling is affected by Sall4 activity. To strengthen their argument, the authors should consider performing in situs to observe expression of targets of Wnt signaling in the embryo, as well as functional manipulation experiments to determine whether (over)activation of Wnt signaling in the cKO embryo is sufficient to rescue the phenotype.

Response: Thank you for this insightful comment, which helped us further investigate whether WNT signaling is affected in *Sall4* cKO.

As suggested, we examined WNT target gene expression. Specifically, we examined expression of *Axin2*, a well-known WNT target gene, by RNAscope. As shown below, the results show downregulation of *Axin2* in paraxial mesoderm in *Sall4* cKO compared to WT, indicating downregulation of WNT signaling in *Sall4* cKO.
We have included this data in Fig. 5E, E’ and described the results in line 269 – 271.

Although not requested, we have also performed additional analysis: RNAscope for *Rspo2*, a

© 2024. Published by The Company of Biologists under the terms of the Creative Commons Attribution License (https://creativecommons.org/licenses/by/4.0/).

positive regulator of WNT signaling, which was downregulated in our RNA-seq analysis (Fig. 5A). As shown below, RNAscope experiments demonstrated that *Rspo2* was downregulated in *Sall4* cKO.

We have included these new data in Figure 5 (Fig. 5C, C', F) and described the results in line 253-254.

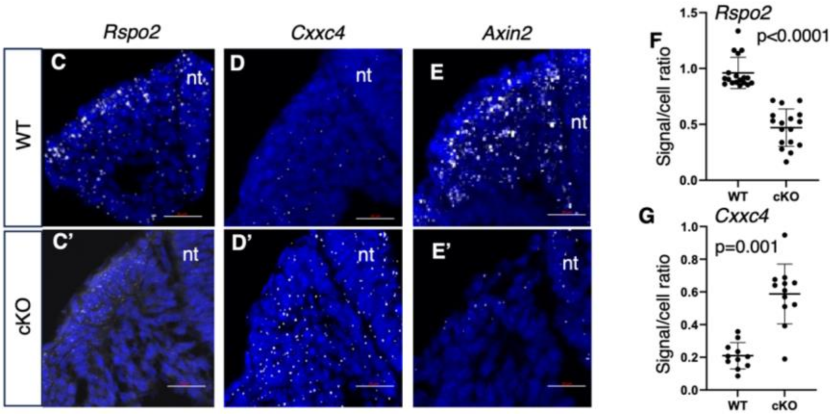

The Reviewer also suggested to test whether overactivation of WNT signaling in *Sall4* cKO embryos is sufficient to rescue the phenotype.

A widely used tool to over-activate WNT signaling involves the use of a *Ctnnb1* exon 3 flox allele (Ctnnb1^{tm1Mmt}). Mice with floxed exon 3 have been used in >500 papers since 1999. As reviewer 3 may know, by crossing *Ctnnb1* exon3 flox mice with a Cre driver, exon 3 is deleted, which results in generation of mutant ß-catenin protein. The mutant form lacks a stretch of Ser/Thr residues, which are phosphorylated for proteosome-mediated degradation of ß-catenin protein. Therefore, ß-catenin protein, generated from exon 3 deleted allele acts as a degradation-resistant, dominant active form, leading to overactivation of WNT signaling.

Since we used *TCre* to knockout *Sall4*, we first assessed the effect of activation of ß-catenin using *TCre*. As shown below, activation of WNT signaling in the *TCre* lineage is deleterious to embryo development and morphogenesis. The mutant embryo exhibited a malformed body, including the posterior trunk and tail. This result indicates that a genetic approach using a *Ctnnb1* ex3 mouse line cannot be used to test whether activation of WNT signaling rescues *Sall4* cKO phenotype.

NOTE: We have removed unpublished data that had been provided for the referees in confidence.

Figure shows a *TCre; Ctnnb1* ex3 embryo (left) and a WT embryo (right) at E10.5. The embryos were hybridized with *Fgf8* probe. An arrow and arrowhead point to the tail tip of mutant and WT embryos, respectively. The *TCre; Ctnnb1* ex3 embryo is malformed, and expression of *Fgf8* is undetectable at the tail tip and limb buds. fl: forelimb bud, h: heart, hl: hindlimb bud.

As an alternative approach, we set up an explant culture system. We dissected the tail (cut at the level of posterior 5th somite) and cultured the tail, similar to Bulusu et al (Dev Cell. 2017 Feb 27;40(4):331-341.e4. PMCID: PMC5337618) with modifications. We cultured the tail in DMEM/F12 with rat serum (20%). We added DMSO (control) or CHIR, which inhibits GSK3ß and hence activates ß-catenin signaling. The culture was done in a humidified chamber with 60% O_2: 35% N_2: 5% CO_2.

We compared expression of *Axin2* and *Lef1*, targets of WNT signaling, in the WT tails, cultured with DMSO or CHIR at a given time point. The result indicates that 4 hours of culture is sufficient to elevate expression of *Axin2* and *Lef1* in CHIR-treated tails compared to DMSO- treated tails (Fig. S6. also shown below), indicating activation of ß-catenin signaling by CHIR.

© 2024. Published by The Company of Biologists under the terms of the Creative Commons Attribution License (https://creativecommons.org/licenses/by/4.0/).

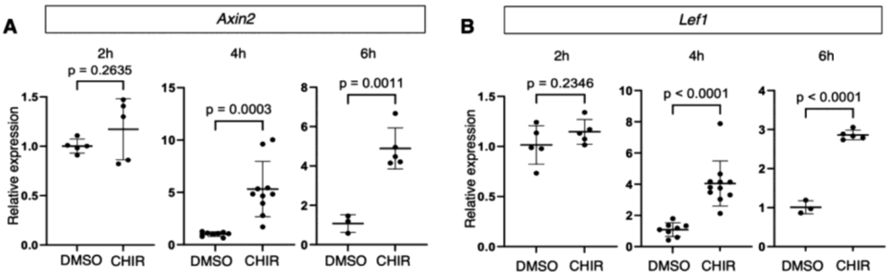

Using this platform, we tested expression of genes expressed in the posterior PSM (*Msgn1*, *Tbx6*), anterior PSM (*Mesp2*, *Ripply2*) and somite (*Tcf15*). First, we cultured WT tail and *Sall4* cKO tail in the presence of DMSO. Consistent with our in situ data in Fig. 1-3, expression of these genes was lower in *Sall4* cKO tail compared to WT tail after 4 hours (Fig. 5H-L). Second, we performed the same experiment with CHIR treatment for 4 hours, which can activate ß- catenin signaling, as described above. After CHIR treatment, the expression levels of these genes were comparable between WT and *Sall4* cKO tail (Fig. 5H'-L'). This result indicates that activation of ß-catenin signaling could rescue expression levels of these genes in *Sall4* cKO embryos and support our notion that ß-catenin signaling acts downstream of *Sall4*.

As shown below, we have included these new data in Fig. S6 and Fig 5H-L'. We have described the results in line 274 - 290.

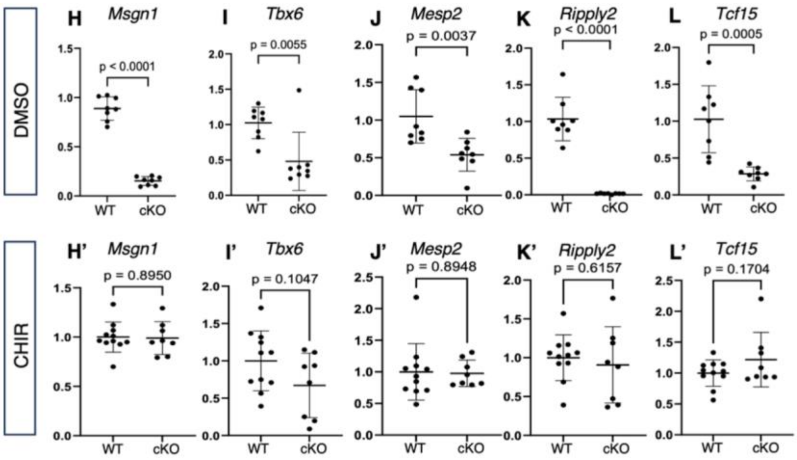

Comment 18: 6. To determine whether differential CTCF peaks are specific to posterior trunk mesoderm the authors compare CTCF occupancy, as inferred from ATAC-seq data, to previously published CTCF ChIP-seq data obtained from mESCs and MEFs. This methodology presents several problems:
a. The authors state that differential CTCF peaks are specific to posterior trunk mesoderm using CTCF peaks mapped to undifferentiated mESCs and MEFs. However, to fully determine whether peaks are specific to mesoderm, the authors should compare their peaks to data generated from mESCs differentiated to neural and mesodermal fates, in addition to undifferentiated mESCs. If the peaks are then solely correlated with the mesodermal cells the authors can more convincingly determine the specificity of CTCF binding peaks to posterior trunk mesoderm.
b. The authors compare CTCF occupancy data inferred from ATAC-seq to CTCF ChIP-seq data. This is not an adequate comparison to determine the specificity of differential CTCF peaks to posterior trunk mesoderm tissue. For the data modalities to be truly comparable, the authors should process previously published mESC and MEF ATAC-seq data using the same TOBIAS method used to calculate inferred CTCF occupancy and compare this inferred occupancy with their data. Conversely, the authors could perform CTCF ChIP-seq analysis of their cHet and cKO samples.

Response: Thank you for the valid suggestions.
We compared our posterior mesoderm data with mESCs to show that that the CTCF signals differ among different cell types. After reading this comment, we appreciate that the reviewer's point

© 2024. Published by The Company of Biologists under the terms of the Creative Commons Attribution License (https://creativecommons.org/licenses/by/4.0/).

that comparison with different mesoderm is important. Regarding the source of the data, we think it is more relevant to use data obtained from different types of mesoderm tissues or neural tissues from mouse embryos.

The reviewer made a very important point that we should process previously published mESCs and MEF ATAC-seq data using the same TOBIAS analysis rather than using CTCF ChIP-seq data. We agree with the reviewer and appreciate that they pointed this out.

To address the critique, we found ATAC-seq data of mESCs (Metzis et al., Cell, 2018 Nov 1;175(4):1105-1118.e17, PMID:30343898, and Tafessu et al., Genome Biol., 2023 Feb 13;24(1):25, PMID: 36782260), and processed them using TOBIAS. As shown below, the posterior trunk mesoderm exhibited different CTCF binding motif occupancy as compared to that in mESCs and MEFs. We have included this new analysis in Fig. S7.

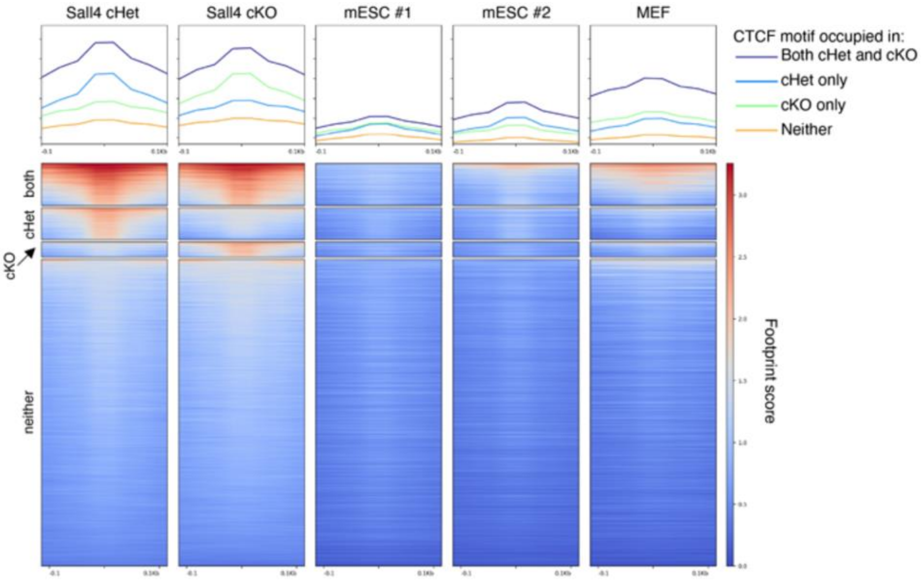

Additionally, we used ATAC-seq data obtained from mouse embryo tissues. More specifically, we used data obtained from T+ cells (mesoderm progenitors) and Sox2+ cells (neural cells) from E8.5 embryos (Koch et al., Dev Cell, 2017 Sep 11;42(5):514-526.e7, PMID: 28826820). We also used our own ATAC-seq data of E9.5-9.75 hindlimb bud cells (Koyano-Nakagawa et al., Nat Commun. 2022 Jul 21;13(1):4221, PMID: 35864091). In addition, we used ATAC-seq data of E9.5 neural tube cells (Metzis et al., Cell, 2018 Nov 1;175(4):1105-1118.e17, PMID:30343898). The result is shown below.

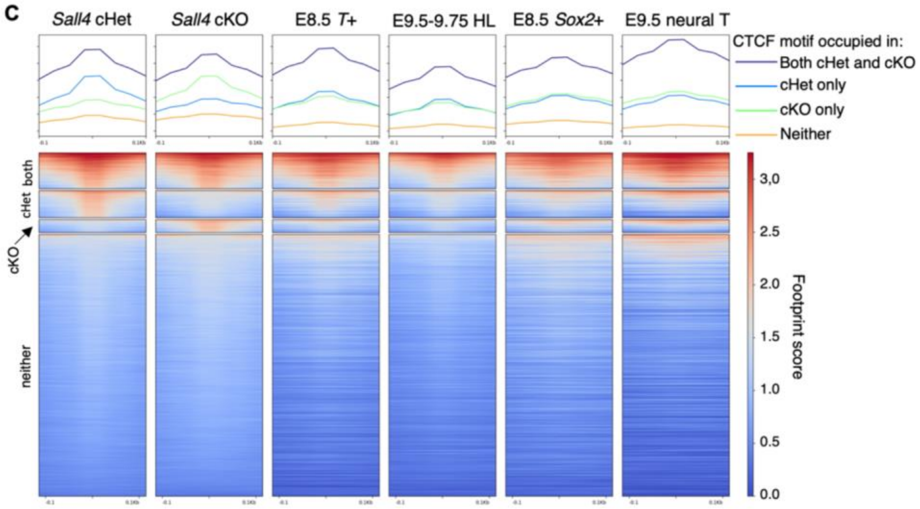

The E8.5 T+ cells are nascent mesoderm, and E9.75 hindlimb bud cells are lateral plate mesoderm-derived cells. These cell types are different mesoderm types from the posterior trunk mesoderm. Moreover, Sox2+ E8.5 cells and E9.5 neural tube cells represent neural cells.

© 2024. Published by The Company of Biologists under the terms of the Creative Commons Attribution License (https://creativecommons.org/licenses/by/4.0/).

The *Sall4* cHet (control) posterior mesoderm showed a subset of occupied CTCF binding motifs that were unoccupied in *Sall4* cKO. GO analysis of the list of these genes show enrichment of neural related biological processes. Given that these sites are unbound in *Sall4* cKO, this suggests *Sall4* dependent occupancy of these CTCF binding motifs. Therefore, CTCF motif occupancy likely contributes to the neural repressive function of *Sall4* in the context of mesoderm tissue.

Importantly, we compared both *Sall4* cKO and *Sall4* cHet CTCF motif occupancy to mESCs, MEFs, and embryo-derived neural tissue and mesoderm tissue, as explained above. We found that the CTCF binding motifs bound in *Sall4* cHet were most strongly occupied in the *Sall4* cHet tissue, suggesting that this footprinting pattern is not equivalent to similar mesoderm tissues nor more distantly related cell types we analyzed. These comparisons support our claim that CTCF footprinting pattern is unique to the posterior trunk mesoderm with respect to other tissues analyzed here.

We have included these new data in Fig. 6C and Fig. S7, and described the result in line 321 - 339.

Minor comments

Comment 19: 1. On page 11, line 279, it is unclear what the term “target genes” is referring to.

Response: We have changed this sentence as below.
Line 342: CTCF is known to function to repress gene expression.

Comment 20: 2. In figure 2, Delta1 is misspelled as Delat1.
Response: We have corrected this mistake.

Resubmission

<u>Second decision letter</u>

MS ID#: DEVELOP/2023/202649

MS TITLE: Sall4 regulates posterior trunk mesoderm development by promoting mesodermal gene expression and chromatin accessibility that promotes WNT signaling and represses neural genes within the mesoderm

AUTHORS: Matthew P. Pappas, Hiroko Kawakami, Dylan Corcoran1, katherine chen, Earl Parker Scott, Julia Wong, Micah D. Gearhart, Ryuichi Nishinakamura, Yasushi Nakagawa, and Yasuhiko Kawakami

ARTICLE TYPE: Research Article

I am happy to tell you that your manuscript has been accepted for publication in Development, pending our standard ethics checks.

<u>Reviewer 1</u>

Advance summary and potential significance to field

The paper uses a conditional KO of Sall4 using TCre, and analyse the effects on posterior trunk mesoderm between E9.5 and E10.5 when lumbar and sacral elements form. Chromatin accessibility is reduced in the mutant, and the expression of genes involved in psm differentiation, and somite formation and differentiation is affected. Wnt pathway components are de-regulated and the

© 2024. Published by The Company of Biologists under the terms of the Creative Commons Attribution License (https://creativecommons.org/licenses/by/4.0/).

expression levels of psm marker genes can be restored using CHIR in an explant essay. The study comprises important new knowledge regarding Sall4 function in the psm.

Comments for the author

My previous comments have been addressed by the authors in their comprehensive response.

© 2024. Published by The Company of Biologists under the terms of the Creative Commons Attribution License (https://creativecommons.org/licenses/by/4.0/).
